# Clinical Significance of MicroRNAs, Long Non-Coding RNAs, and CircRNAs in Cardiovascular Diseases

**DOI:** 10.3390/cells12121629

**Published:** 2023-06-14

**Authors:** Desh Deepak Singh, Youngsun Kim, Seung Ah Choi, Ihn Han, Dharmendra Kumar Yadav

**Affiliations:** 1 Amity Institute of Biotechnology, Amity University Rajasthan, Jaipur 303002, India; ddsbms@gmail.com; 2 Department of Obstetrics and Gynecology, College of Medicine, Kyung Hee University, Seoul 02447, Republic of Korea; chacha0725@naver.com; 3 Division of Pediatric Neurosurgery, Pediatric Clinical Neuroscience Center, Seoul National University Children’s Hospital, Seoul 08826, Republic of Korea; aiipo@snu.ac.kr; 4 Plasma Bioscience Research Center, Applied Plasma Medicine Center, Department of Plasma Biodisplay, Kwangwoon University, Seoul 01897, Republic of Korea; 5 Department of Pharmacy, Gachon Institute of Pharmaceutical Science, College of Pharmacy, Gachon University, Incheon 21924, Republic of Korea

**Keywords:** cardiovascular disease, microRNAs, long noncoding RNA, diagnosis, ncRNAs, therapy

## Abstract

Based on recent research, the non-coding genome is essential for controlling genes and genetic programming during development, as well as for health and cardiovascular diseases (CVDs). The microRNAs (miRNAs), lncRNAs (long ncRNAs), and circRNAs (circular RNAs) with significant regulatory and structural roles make up approximately 99% of the human genome, which does not contain proteins. Non-coding RNAs (ncRNA) have been discovered to be essential novel regulators of cardiovascular risk factors and cellular processes, making them significant prospects for advanced diagnostics and prognosis evaluation. Cases of CVDs are rising due to limitations in the current therapeutic approach; most of the treatment options are based on the coding transcripts that encode proteins. Recently, various investigations have shown the role of nc-RNA in the early diagnosis and treatment of CVDs. Furthermore, the development of novel diagnoses and treatments based on miRNAs, lncRNAs, and circRNAs could be more helpful in the clinical management of patients with CVDs. CVDs are classified into various types of heart diseases, including cardiac hypertrophy (CH), heart failure (HF), rheumatic heart disease (RHD), acute coronary syndrome (ACS), myocardial infarction (MI), atherosclerosis (AS), myocardial fibrosis (MF), arrhythmia (ARR), and pulmonary arterial hypertension (PAH). Here, we discuss the biological and clinical importance of miRNAs, lncRNAs, and circRNAs and their expression profiles and manipulation of non-coding transcripts in CVDs, which will deliver an in-depth knowledge of the role of ncRNAs in CVDs for progressing new clinical diagnosis and treatment.

## 1. Introduction

**Figure 1:**
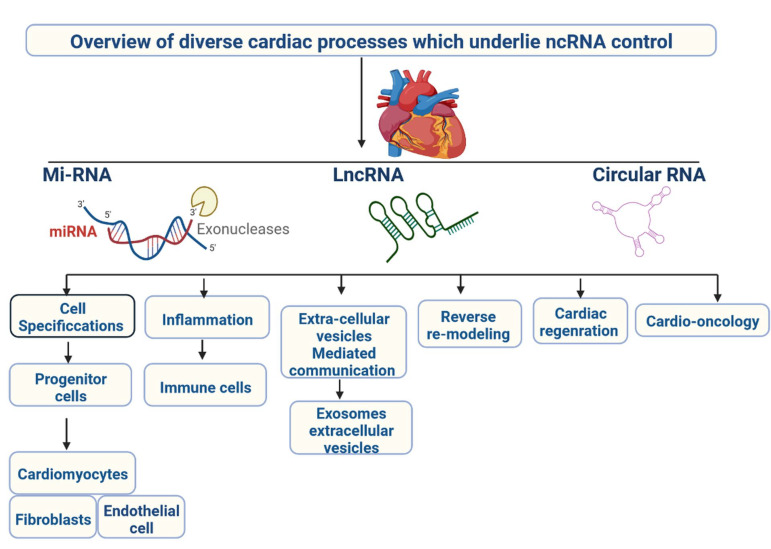
Overview of various cardiac processes controlled by ncRNA.

CVDs are caused by a reduced flow of oxygenated blood in the human body. CVDs are a group of diseases, including ACS, MI, AS, CH, RHD, MF, PAH, and ARR [1,2]. CVDs are the major cause of death worldwide, according to the World Health Organisation; 17.9 million deaths were reported in 2019, accounting for 32% of all deaths at the global level. Early diagnosis and targeted treatment of CVDs remain challenges [3,4]. Current treatment options are available, with their limitations, and can reduce disease development. Novel treatment options are required to target cellular events during disease progression to facilitate the timely management of the patient’s clinical conditions [5]. Multiple coding genes are involved in the development of CVDs. In recent years, it has been discovered that non-coding RNA (ncRNA) regulates disease development [6]. The ncRNAs are 200-nt base sequences that regulate the genetic, epigenetic, and cell signalling mechanisms, as well as gene expression (Figure 1) [7]. ncRNAs are used as biomarkers for diagnosis and treatment due to their involvement in disease severity. In recent years, ncRNA has been investigated in CVDs [8]. ncRNAs have great importance in clinical applications and are classified into various categories, including miRNAs, lncRNAs, and circRNAs [9]. SiRNAs can be used to target ncRNAs. RNAi-mediated siRNAs are highly adaptable and are used to silence their mRNA’s protein-encoding gene. MiRNAs are 22-nucleotide RNA molecules that regulate cell signalling and downregulate the expression of specific genes by modifying the translation process [10].

**Figure 2:**
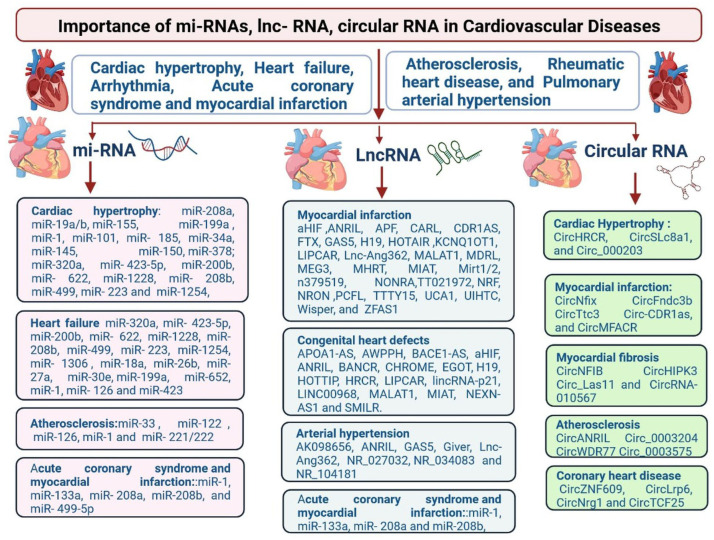
Regulations of miRNA, lncRNA and circRNA in cardiac hypertrophy (CH), heart failure (HF), rheumatic heart disease (RHD), acute coronary syndrome (ACS), myocardial infarction (MI), atherosclerosis (AS), myocardial fibrosis (MF), arrhythmia (ARR), and pulmonary arterial hypertension (PAH).

Clinically relevant non-coding RNAs in CVDs are classified by using various tools and techniques [11,12,13,14]. The ncRNAs highlighted in Figure 2 are present in myocardial infarction, coronary artery disease, heart failure, and arterial hypertension [11,12,13,14]. The majority of the circulating miRNAs are derived from blood cells, with others from various tissues, such as the heart [12,13,14,15]. Regulations of lncRNA in cardiovascular disorders and their clinical significance are identified in acute coronary syndrome and myocardial (miR-1, miR-133a, miR-208a, miR-208b, miR-499p), arteriosclerosis (miR-33a, miR-122, miR-126, miR-1, miR-126, miR-1, and miR-221/222), cardiac hypertrophy (miR-208a, miR- 19a/b, miR-155, miR-199a, miR-1, miR-101, miR-185, miR-34a, miR-145, miR-150, miR-178, miR-320a, miR-425b), and heart failure (miR-320a, miR-4235p, miR-4235p, miR-200b, miR-4235p, miR-4235p, miR-4235p, miR-200b, miR-200b, miR-622, miR-1228, miR-200b, miR-200b, miR-499, miR-499, miR-223, miR-499, miR-200b, miR-499, miR-223, miR-1306, miR-18a, miR-26b, miR-27a, miR-26b, miR-26b, miR-26b, reduced levels of miR-126, members of the miR-17-92 cluster, inflammation-related miR-155, and smooth muscle-enriched miR-145 in patients with CAD compared with healthy controls [15,16,17,18,19,20,21,22,23,24,25,26,27,28,29,30,31,32,33,34,35,36,37,38,39,40,41,42,43,44,45,46,47,48,49,50,51,52,53,54,55]. In contrast, cardiac muscle-enriched miRNAs (miR-133a, miR-208a) tended to be higher in patients with CAD [56,57,58,59,60]. There is additional evidence that cardiac risk factors affect circulating miRNA levels. It was shown that patients suffering from prevalent diabetes have significantly decreased levels of miR-20b, miR-21, miR-24, miR-15a, miR-126, miR-191, miR-197, miR-223, miR-320, and miR-486, but a modest increase of miR-28-3p. In univariate and multivariate analyses, 29 MiR-126 data points were confirmed in 822 patients [61,62,63,64,65,66,67,68,69,70]. In patients with diabetes, the reduction of miR-126 was confined to circulating vesicles in plasma [71,72,73,74]. The human circRNAs 0037911, _0126991, and _0005870 seem to be the best candidates as hypertension biomarkers. CircRNAs 0037911 and 0126991 were found to be highly regulated in the blood of hypertensive patients, while circRNA 0005870 was downregulated. Taken together, these circRNAs seem to be involved in vascular endothelial dysfunction and, consequently, the development of arterial hypertension [7,8,9,10]. CircRNAs are involved in cardiac hypertrophy (CircHRCR, CircSLc8a1, and Circ_000203), myocardial infarction (CircNFIB, CircHIPK3, and CircRNA10567), cardiac hypertrophy (CircHRCR, CircSLc8a1, and Circ_000203), arteriosclerosis (CircANRIL, Circ-0003204, CircWDR77, CircTCF25), and in coronary heart disease (CircZNF609, CircLrp6, CircNrg1, and CircTCF2) (Figure 2) [11,12,13,14,15].

Current investigations are still in the beginning stages, and the classification of miRNAs, lncRNAs, and circRNAs and their regulatory mechanisms and functions are still not well known [11]. Therefore, recent investigations clearly establish essentially novel therapeutic targets based on the unusual structure of RNA to address current challenges in patients with CVDs [12]. Till now, only a small amount of non-coding protein transcript has been identified and explored for its clinical applications in CVDs. There are various major non-coding therapies for CVDs in the pipeline (Table 1). MiRNA, lncRNA, and circRNA have clinical significance in cardiovascular disease (Figure 2) [12]. Here, we discuss the biogenesis, identification, and regulatory mechanisms of miRNAs, lncRNAs, and circRNAs, as well as their clinical importance, limitations, expression profile regulation, and manipulation in CVDs, including CH, HF, ARR, ACS, MI, AS, RHD, pulmonary arterial disease (PAD), and hypertension. Furthermore, we will discuss clinical investigations of cardiovascular non-coding RNA therapies and future perspectives.

## 2. miRNAs and CVDs 

**Figure 3:**
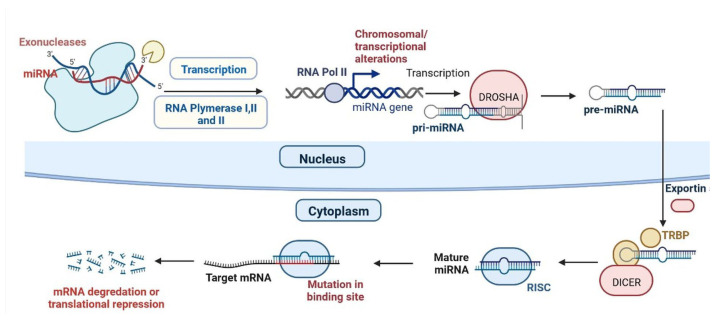
Synthesis and mechanism of miRNA.

The miRNA lin-4 was identified in 1993 in Caenorhabditis [71]. The synthesis of miRNA occurs in the nucleus and is transcribed by RNA polymerase II into coding and noncoding, capping polyadenylated pri-miRNAs. The pre-miRNA produces a hairpin-like structure and is timed by the Drosha nuclear enzyme, then transported to the cytoplasm [12]. The DICER removes the terminal loop of the pri-miRNAs, resulting in a 20-25 nucleotide base pair dsRNA complex. The dsRNA, as attached to the miRNA-linked RISC (RNA-induced silencing complex), targets mRNAs and results in mRNA de-adenylation and translational repression (Figure 3) [13]. In vivo and in vitro studies have shown that miRNA plays a critical role in the regulation of CVDs such as CH, HF, ARR, ACS, MI, AS, RHD, and PAH (Table 2) [14].

Myocardial hypertrophy (MH) is caused by the development of CVDs, including stenosis of the heart valve and hypertension, and causes HF and death [75]. Several miRNAs, including miR-208a, miR-19a/b, miR-34a, miR-145, miR-150, miR-378, and others, are involved in the development of MH [76]. MiR-378 is an anti-MH miRNA and regulates the Igf1r (insulin-like growth factor receptor), Grb2 (growth factor receptor binding protein 2), and Ksr1 (Ras kinase inhibitor 1) [77]. MiR-185 regulates cardiac cell proliferation and is related to the signal transduction mechanism. MiR-34a regulates the Agt9a gene, which is involved in autophagy. The transcription activator p300 is regulated by miR-150 [78]. MiR-1 is involved in the growth and development of cardiomyocytes by reducing the expression of GATA-binding protein 4 (GATA4) and calmodulin Mef2a, which regulate the calcium signal pathway and protein expression and could be targeted for diagnosis and therapy [79].

### 2.1. miRNAs and HF

HF is caused by a failure of the regulatory mechanism in the heart [80]. Many different forms of miRNA, such as miR-320a, miR-423-5p, miR-200b, miR-622, miR-1228, miR-208b, miR-499, miR-223, miR-1254, miR-1306, miR-18a, miR-26b, miR-27a, miR-30e, miR-106a, miR-199a, are crucial in the development of HF conditions [81]. Early hypertrophic growth in the left ventricle may be caused by miR-125b and lead to HF. The expression of brain natriuretic peptide (BNP) is regulated by miR-200b, miR-622, and miR-1228. HF may also be caused by increased expression of miR-208b and miR-499. These miRNA regulations in HF could be targeted for diagnosis and therapeutic approaches [82].

### 2.2. Arrhythmias

Arrhythmias (AR) are mainly caused by imbalances of the ion channel and dysregulations of conduction in cardiac muscles. Atrial fibrillation (AF) is a severe AR observed in CVDs that can lead to HF, stroke, and death [83]. There are various types of miRNA involved in the development of AR in CVD patients, including miR-664, miR-133, miR-590, miR-130a, miR-21, miR-208b, miR-483, miR-1, and miR-150. In addition, the AF is controlled by the miRNAs miR-328, miR-2, miR-664, miR-483, miR-133, miR-1, miR-208b, miR-590, miR-328, and miR-223 [84]. The overexpression of miR-130a is linked with cx43 (protein connexin 43). MiR-150 regulates the platelet count in patients with AF, which plays a major role in fibrosis and inflammation and is involved in the development of AF [85].

### 2.3. miRNAs and ACS and MI

ACS (acute coronary syndrome) is developed by reduced blood flow in the heart, an immediate blockage of the coronary arteries, and localized heart necrosis, all contribute to the development of AMI (acute myocardial infractions) [86]. AMI patients have a high level of miR-1 expression. MiR-1, miR-133a, and miR-208a levels have been found to be higher in AMI patients. Cardiac arrest is regulated by miR-208b and miR-499-5p in patients with coronary artery bypass grafting [87]. These two miRNAs are expressed by dysregulated cardiac muscles. A reduced level of expression has been shown in AMI patients. The expression profile of all these miRNA regulations can be used for early diagnosis and treatment [88]. High miR-208 expression levels have been observed in a mouse model with AMI. High-throughput analysis of miRNA expression in patients with AMI can be explored further for sensitive and specific early diagnosis and treatment [89].

### 2.4. miRNAs and Atherosclerosis

The miRNA plays an important role in the generation of atherosclerosis by vascular angiogenesis, endothelial dysfunction, lipid accumulation, local inflammation, calcification, thrombosis, and endothelial dysfunction [90]. Play important in the development of CAD (coronary artery disease), causes significant death at global level. Expression profile of miRNA has been investigated in patients with AS. MiR-33 regulates the AS disease progression by involving the inflammatory response, cell cycle progression, lipid metabolism, and proliferation [91]. In patients with AS, miR-122 is substantially expressed. MiR-122 controls the levels of high-density lipoprotein (HDL) and low-density lipoprotein (LDL).Leukocyte aggregation on endothelial cells is triggered by miR-126-mediated upregulation of VCAM-1 (vascular cell adhesion molecule-1) [92]. Mi-R1 regulates the signalling pathways for MLCK (Myosin Light Chain Kinase) and ERK (Extracellular Signal-Regulated Kinase). MiR-221 and miR-222 control the growth and development of vascular smooth muscle cells (VSMCs). In patients with AS, there is generally less miR-126, miR-1, and miR-221/222 expression [93].

### 2.5. miRNAs and RHD

RHD lesions have primarily been found in the mitral valve. RHD tissue and plasma samples have significant levels of miRNA-1299 and miRNA-1183 expression. MiR-328-3p is found in RHD and AF (atrial fibrillation) patients [94]. MiRNA-432 expression levels have been found to be lower in RHD patients. All these microRNAs could be used for early diagnosis. Further investigations are needed to find out more about miRNA regulations in RHD [95].

### 2.6. LncRNAs and Cardiovascular Diseases

**Figure 4:**
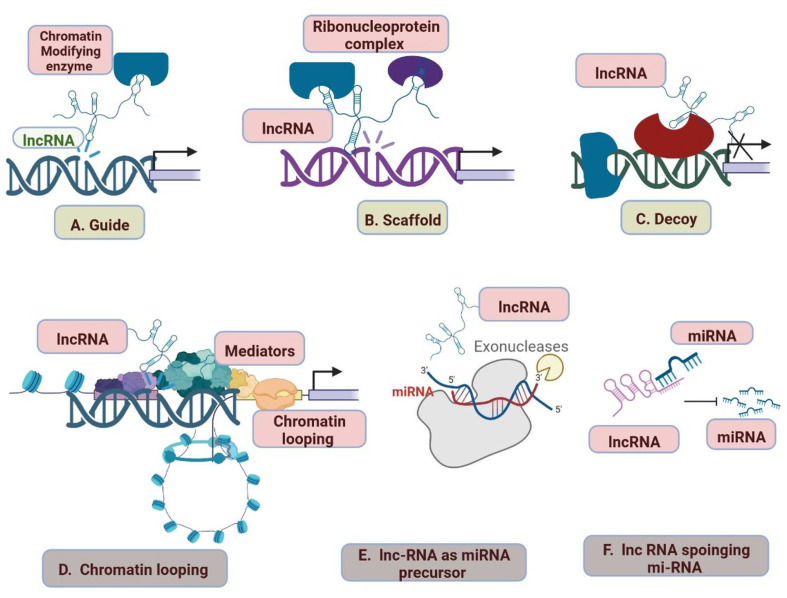
lncRNA mechanisms of action. (**A**) Guide lncRNAs activate or repress gene expression through relocalization of regulatory factors. (**B**) Scaffold lncRNAs aid in the formation of Ribonucleoprotein (RNP) complexes. (**C**) Decoy lncRNAs remove the regulatory factor bound to the genome, thereby terminating its regulation. (**D**) lncRNAs sponge the miRNAs, thus inhibiting the miRNA-mediated gene repression. (**E**) miRNA precursor lncRNAs function as primary miRNA precursors that are processed into mature miRNAs. (**F**) lncRNA transcription from regulatory regions of the genome initiates long-range gene regulation.

**Figure 5:**
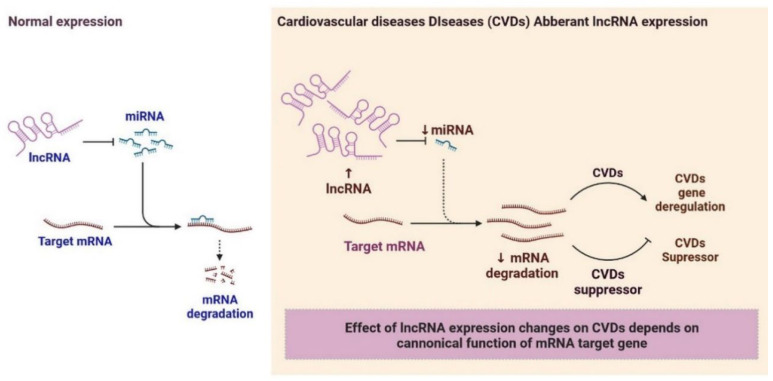
lncRNA-miRNA Gene Expression: Effect of lncRNA expression changes on CVDs depends on canonical function of miRNA target gene.

LncRNAs are more complex and heterogeneous in nature in comparison to miRNAs, which regulate gene expression. LncRNAs are involved in CVDs and categorized into various classes based on their structure and functions, including bidirectional lncRNAs, enhancer lncRNAs, sense lncRNAs, antisense lncRNAs, intergenic lncRNAs, and intron lncRNAs [96]. The gene expression level is changed by the interactions of lncRNA with DNA, RNA, proteins, elements of the chromatin modification complex, and transcription factors. Guided lncRNAs can either activate lncRNA processes or suppress gene expression by delocalizing regulatory elements [97,98,99]. Ribonucleoprotein (RNP) complex formation involves the scaffold lncRNAs (Figure 4). The lncRNAs serve as primary miRNA precursors that are converted into mature miRNAs while the miRNA precursor is suppressed. Long-range gene regulation begins when the lncRNA activates transcription from regulatory areas of the genome. LncRNAs interact with miRNAs and disrupt the RNA molecules’ regulatory system (Figure 5) [97]. lncRNAs also act as a maternal or paternal genomic imprinting expression and help in the development of organisms [100,101]. Regulations and clinical importance of lncRNA in cardiovascular are shown in Table 3.

The integration of various types of cells, the vascular system, and blood vessels are all involved in the generation of the heart [98]. lncRNAs, also known as super-enhancer lncRNAs (SE-lncRNAs), control transcription at the tissue and cell levels. MyoD is an important transcription factor that involves muscle cell differentiation along with other core transcription factors [99]. The CE (core enhancer element) is produced by CERNA, which acts as a positive feedback regulator. It has been recently observed that various types of lncRNA are involved in the development of CVDs, including CHRF, Myh7, LIPCAR, MIAT, Carl, LIPCAR, ASB9P1, RP11-218 M11.6, G078882, G064270, G000678, G030563, H19, TUG1, PFL, MIAT, AK081284, HOXA11-ASz, NRON, and GAS5 [96]. H19 is expressed during embryogenesis and CVD but is repressed after birth. miRNA-675 acts as a negative regulator in cardiac hypertrophy. miR-675-3p and miR-675-5p are upregulated in cardiac hypertrophy [100]. Some pro-hypertrophic factors are also involved in CH and are mediated by Ca/calmodulin-dependent protein kinase IIδ (CaMKIIδ). The lncRNA–miRNA–mRNA axis can be a potential target for therapeutic approaches [101]. All these investigations have confirmed that lncRNAs play major roles in cardiovascular biology and diseases (Table 3) [102].

## 3. Clinical Significance of lncRNAs in Cardiovascular Diseases

### 3.1. Arterial Hypertension

There are different kinds of lncRNAs that control vascular tone to control the pathophysiology of AH (Table 3). LncRNAs contributor AH controls VSMC dysfunction, and miRNAs 221 and 222, which control lnc-Ang362, control the growth of VSMCs [158,159]. The lncRNAs NR_027032, NR_034083, and NR_104181 act as biomarkers for disease diagnosis in AH. Differential expression of lncRNAs in rats exposed to a peptide hormone (Angiotensin-II) with a variety of functions, including inflammation, fibrosis, vasoconstriction, and hypertrophy/hyperplasia [140]. The silencing of lnc-Ang362 reduced the expression of miRNAs, which led to a reduction in VSMC proliferation. The lncRNA GAS5 (growth arrest-specific 5) regulates vascular remodelling in hypertension and is expressed primarily in ECs and VSMCs [160]. The proliferation of VSMC lncRNAs NR4A3 and AK098656 induces the oxidative stress-induced proliferation of VSMCs. CDKN2B-AS1 antisense lncRNA susceptibility to the development of AH [161].

### 3.2. Coronary Heart Disease

Plaque formation in CVDs is caused by a chronic inflammatory process that narrows the vessels and reduces blood flow, resulting in atherosclerosis and ischemia, which lead to the development of CHD (Table 3) [162]. There are various lncRNAs that are linked to the pathophysiology of CHD and act as biomarkers, including MIAT, MALAT1, ANRIL, LIPCAR, MALAT1, MIAT, and SMILR. HOTTIP, a lincRNA-p21, regulates cell proliferation and apoptosis (Table 3) [163]. The lncRNA BANCR is associated with CHROME (the cholesterol homeostasis regulator of miRNA expression). lncRNA NEXN-AS1 is involved in the mitigation of atherosclerosis. All these RNAs may be targeted for the development of diagnosis and treatment [164].

### 3.3. Acute Myocardial Infarction

AMI is caused by a blockage in blood flow, less oxygen in the blood, and metabolic disorders. There are various lncRNAs involved in potential signalling in AMI (Table 3) [165]. In a rat model, the lncRNA MIAT is involved in myocardium degeneration and regulates adverse heart remodelling [165]. H19 is involved in autophagy, HOTAIR is involved in the cardioprotective activity and sponging with miRNA, KCNQ1OT1 in left ventricle dysfunctions, MALAT1 regulates cardiomyocytes, MDRL in mitochondrial dysfunctions, MEG3 and MEG3 in cardiomyocyte apoptosis, MIAT regulates CH, Mirt1/2 regulates cardiac remodelling [102], NONRATT021972 regulates cardiac activity, PCFL regulates cardiac fibroAll these lncRNAs have shown great importance in the regulation of pathological conditions, diagnosis, and treatments.

### 3.4. Heart Failure

In chronic conditions, the heart does not pump systolic and diastolic properly, which may increase shortness of breath, fatigue, inflammation, and a fast heartbeat [166]. There are various lncRNAs involved in HF, including lncRNAs MHRT, CHAER, CHRF, APF, CARL, aHIF, MIAT, and CHAST [167]. MHRT is a cluster of specific RNA that plays an important role in maintaining the physiological conditions of the heart. Reduced expression of MHRT was observed in HF conditions, isoforms of MHRT, Myh6, and Myh7 are involved in HF [144]. During a cardiac stress response, Brg1 regulates the homeostasis of the heavy chain from -myosin to -myosin. The overexpression of CHRF induces a pathological process and induces apoptosis. The lncRNA CHAER is involved in cardiac remodelling and is expressed during HF in the heart of mice [147]. The long noncoding RNA PCR2 has shown cardioprotective properties in animal models. APF regulates autophagy, CARL regulates angiogenesis, aHIF regulates apoptosis, and MIAT regulates the cardiac fibrosis process in MI [145]. Vascular remodelling in arterial hypertension is regulated by GAS5 and AK0986656 lncRNAs. Studies on all these lncRNAs under investigation could be major targets for diagnosis and treatment before HF (Table 3) [145].

## 4. Circular RNA and Cardiovascular Diseases

**Figure 6:**
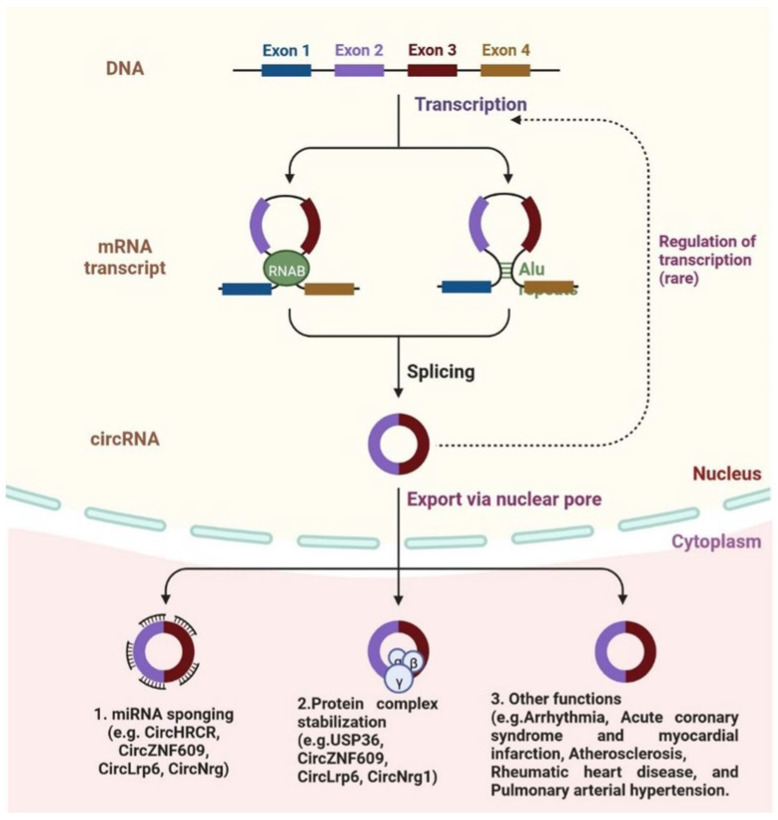
circRNA biogenesis is shown schematically. The production of circRNAs is possible by a direct backsplicing mechanism in which intronic reverse complementary sequences or RNA-binding protein (RBP) pairing, which brings the sequences close together to promote circularization and drive circularization. CircRNAs can also be produced by a mechanism called lariat-driven, which is characterized by a lariat structure with flanking sequences that help produce circRNAs. circRNAs are exported via the nuclear pore, act as mRNA sponges (CircHRCR, CircZNF609, CircNrg), stabilize protein complexes (USP36, CircLrp6, CircNrg1), and are involved in other functions.

CircRNAs are SS-RNAs, covalently closed and forming a loop; the 3′ and 5′ ends of RNA molecules are joined [168]. CircRNAs regulate gene expression and code for proteins; circRNA has been linked to some diseases, such as cancer, diabetes, cardiovascular disease, and others (Figure 6) [169]. CircRNAs play an important role in the regulation of molecular mechanisms in the cardiovascular system (Table 4). cZNF292 is the first-identified circRNA in endothelial cells (ECs) and controls angiogenesis [170]. CAD and SMCs (smooth muscle cells) are regulated by circRNA ANRIL; it also regulates proliferation and apoptosis by interfering with the rRNA (ribosomal RNA) maturation process [171]. The vascular SMCs are regulated by circLrp6; another circFoxo3a is involved in cardiac functions, and cardiac muscles are required by circFndc3c after MI. Heart-related circular RNA (HRCR) circRNA and CDR1 act as miRNA sponges [172]. CDR1 is involved in the development of post-myocardial infractions in mice via sponging miR-7. CDR1 is associated with the LV (left ventricular) and RV (right ventricular) and is used as a clinical biomarker for disease diagnosis. CircYOD-1 is used as a biomarker for coronary artery disease [173]. Some circRNAs are observed in paediatric patients with CHD, including HSA circRNA 004183, HSA circRNA 079265, and HSA circRNA 105039, among others. Three circRNAs, namely DNAJC6, TMEM56, and MBOAT2, are identified in patients with hypertrophic cardiomyopathy (HCM). All these biomarkers could be used for diagnosis [174].

### 4.1. circRNA in Atherosclerosis

VSMCs and VEC are involved in the pathogenesis of AS; the circRNA-miRNA-mRNA axis plays a major role in the development of AS [193]. The circRNA hsa-circ-0054633 protects the endothelial cells or tissues from injury by hyperglycemic conditions. Overexpression of circRNA hsa-circ-0054633 induces regulation of miR-218 and regulates the miR-218/HO-1 and miR-218/ROBO1 axis. By regulating the expression of the miR-186/HIF-1 axis, hsa-circ-0010729 increased apoptosis and prevented the proliferation of hypoxia-induced HUVEC (human umbilical vein endothelial cells), reducing endothelial injury [194]. The circRNA circWDR77 prevents the proliferation and migration of VSMCs by regulating miR-124 and FGF-2 (fibroblast growth factor 2). The miR-661/SYK axis activates proliferation and migration by circRNA circ-RUSC2 [195]. All these regulations of circRNA could be major targets for early diagnosis and treatment.

### 4.2. Coronary Heart Disease (CHD)

The CHD is regulated by various types of circRNAs, which play an important role in pathological mechanisms, including circ-SATB2, SM22α, circ-171, and circ-624. Circ-SATB2 has been reported to be upregulated in the proliferative mode of VSMCs and downregulated in contractile VSMCs [169]. There are various lncRNA types that regulate vascular tone to regulate the pathophysiology of AH. Giver AH, a lncRNA, regulates VSMC dysfunction, and miRNAs 221 and 222, which regulate lnc-Ang362, regulate VSMC expansion [196]. These investigations suggest that circ-RNA plays a major role in CHD and could be used as a diagnostic marker.

### 4.3. Cardiac Ischaemia/Reperfusion (I/R) Injury and Myocardial Infarction (MI)

Treatment of MI is based on the pathological conditions of cardiac ischemia (CI) and reperfusion; CI leads to mitochondrial dysfunction and induces apoptosis of cardiac muscle cells. Recent research has shown that the circRNA–miRNA–mRNA axis plays an important role in CI and R injuries and MI [197]. CircumNCX1 increases ROS expression in CI and R injury and regulates apoptosis via the circNCX1–miR-133a-3p–CDIP1 axis [198]. The overexpression of CDIP1 also induces the apoptosis process. The sponging miR-652-3p is regulated by circ-MFACR, which induces the expression of MTP18, which leads to mitochondrial dysfunction and apoptosis of cardiomyocytes [199].

### 4.4. Heart Failure (HF)

HF is mainly caused by MI and cardiac hypertrophy (CH). The development of CH is prevented by circ-HRCR via endogenous regulation of miR-223. Expression of another circRNA, circ-Nfix, induces the regeneration capacity in mice after MI by using the miR-214/Gsk3β axis [200]. The increased expression of circSLC8A1 has been observed in CH, but at the same time, the expression of miR-133a has been downregulated. The diagnosis and therapeutic targets of [201] could be a promising approach to treating CH.

### 4.5. Cardiomyopathies (CM)

CM is caused by a group of cardiac diseases (CD), which dysregulate cardiac functions and lead to HF and high morbidity and mortality reported at the global level [202]. The high-throughput sequencing analysis data has been investigated in patients with dilated DCM (dilated cardiomyopathies), and 9585 circRNAs were differentially regulated. One of the most prevalent circRNAs, CircSLC8A1, is crucial for CM differentiation, cardiac development, and homeostasis [203]. The pathophysiology of CMs is greatly influenced by the cardiac-specific expression of circALPK2, circSPHKAP, circCACNA1D, and circSLC8A1 and acts as a diagnostic biomarker [169]. The highly expressed circRNAs in DCM were regulated by the BTBD7, NHLRC2, FAT1, LYPLAL1, DHX40, DHX40, ICA1, TTN, and PKN2 genes [183]. The circRNAs involved in DCM are described in Table 4. All these investigations have proven the importance of circRNAs in CM [192].

### 4.6. circRNAS in Cardiac Regeneration

Various types of circRNAs are involved in cardiac regeneration (CR), reprogramming, and proliferation of the myocardium (Table 4). The circNfix regulates proliferation and angiogenesis and improves the cardiac function of cardiomyocytes in a mouse model [204]. Overexpression of CircCDYL promotes the CR, inhibition of CircCDYL induces the proliferation of fibroblasts, CircHIPK3 promotes cellular growth, and CircHIPK3 induces angiogenesis in the myocardium in mice [204]. The diagnosis and therapeutic importance of circRNAs in CVDs need to be explored further.

## 5. Clinical Investigations of Cardiovascular Non-Coding RNA Therapies

Clinical investigations of cardiovascular non-coding RNA therapies are under investigation, including CM hypertrophy, altered excitation–contraction coupling, cell death, interstitial fibrosis, and microvascular rarefaction [10,11,12,13,14,96,193,205]. The novel ncRNA therapeutics under clinical investigation are shown in Table 5 [206]. In order to silence the miRNAs responsible for cardiac hypertrophy, a typical strategy has been to use 2′-OMe-modified (*2*′-*O-Methyl* (*2*′-*OMe*)-4′-thioRNA) antagomiRs or LNAs (locked nucleic acid-modified), with the earliest evidence suggesting in vivo silencing originally stated in mice [207,208]. Several miRNAs that operate on either CMs or fibroblasts have been demonstrated to suppress hypertrophy and remodelling during experimental HF in mice [208]. These include miR-133, miR-199a-5p, miR-21, miR-23a, miR-24, mir-29, miR-34a, and miR-25. Other studies reported success with LNA ASOs, e.g., against mir-34, miR-652, miR-208a, miR-154, miR-29, and miR-21 in pigs. Other cell-type-specific strategies might also be successful. Mice with chronic pressure overload had less cardiac inflammation, hypertrophy, and dysfunction after leucocyte-expressed miR-155 was inhibited [209]. An LNA that targets miR-26a was intravenously administered, and this quickly stimulated angiogenesis, decreased the size of MIs, and enhanced heart performance [207,208]. Osteopontin, a protein that encourages fibrosis and hypertrophy, was the target of an RNA aptamer that prevented or reversed pressure overload-induced coronary artery disease in mice [207]. The inhibition of endothelial miR-24 reduced the size of MI by decreasing endothelial apoptosis. The lncRNA Chast was effectively silenced by GapmeR, which prevented or reduced stress overload-induced pathological cardiac remodelling as one of the first examples of therapeutic lncRNA targeting in the cardiovascular sector [208]. The lncRNA Meg3 reduces cardiac fibrosis and improves diastolic function after chronic pressure overload. Wisper is a lncRNA that is actively involved in preventing heart dysfunction and fibrosis induced by MI, and lncRNA H19 prevents the growth of pulmonary arterial hypertension or aortic aneurysms [205,206]. Various ncRNA medications are under development to address various aspects of hyperlipidemia as a result of the ease with which the liver can be targeted [206]. The LPA (Lipoprotein A) is a significant target that is currently unaffected by small-molecule medications but is a major carrier of oxidized phospholipids in human plasma and a causal risk factor for atherosclerotic CVD and aortic stenosis [205]. Apo(a), the LPA gene’s product, is covalently linked to ApoB-100 in lipoprotein(a). Pelacarsen (formerly AKCEA-APO(a)-LRx), an ASO-targeting LPA mRNA, decreases Lp(a) levels by up to 80% and is well tolerated aside from injection-site responses. Aantisense oligonucleotide targeting LPA mRNA (which encodes the main Lp(a) constituent, apolipoprotein(a)) conjugated with triantennary N-acetylgalactosamine to directs the therapy [206]. It is administered subcutaneously two to four times per week. Patients with CVD are now participating in Phase 3 trials for it (NCT04023552) [206]. Olpasiran, a GalNAc-conjugated siRNA that lowers Lp(a), is currently being studied in Phase 2 (NCT04270760), and SLN360, another GalNAc-conjugated siRNA, is being tested in Phase 1 (NCT04606602). Angiopoietin-like 3 (ANGPTL3) is another intriguing hyperlipidemia target, as evidenced by research showing that loss-of-function variations are linked to significantly lower LDL-cholesterol and triglyceride levels and a decreased risk of CHD [206]. The circulating lipoprotein lipase and endothelial lipase inhibitor ANGPTL3, which is mostly made in the liver, influences the uptake of muscle-free fatty acids, adipose tissue lipogenesis, and LDL and residual cholesterol by the liver [206,207]. The latter effects are significant since they are independent of the LDL receptor, indicating that ANGPTL3 reduction should be helpful in FH patients. with mutations in the LDL receptor. In a Phase 2b study (NCT04516291), Vupanorsen, a GalNAc-modified ASO-targeting ANGPTL3 mRNA, was tested in diabetic patients with hepatic steatosis and hypertriglyceridemia [205,206,207,208]. It reduced triglycerides, ApoC3, and residual cholesterol by 38–58%. Finally, ARO-ANG3, a GalNAc-conjugated siRNA that targets ANGPTL3 as well, has been tested in a larger Phase 2 trial (NCT04832971) after being evaluated in a smaller open-label study in heterozygous FH87 [206]. Inhibition of miR-92a with antisense oligonucleotides improves wound healing, speeds up re-endothelialization, and avoids endothelial dysfunction and atherosclerosis in murine and porcine models after MI and hind limb ischaemia. In pigs, administration of an anti-miR-92a LNA ASO via catheter significantly decreased infarct size and enhanced heart function. An LNA ASO targeting miR-132-3p for patients with HF was also reported to have first-in-human data [206].

## 6. Future Prospective

Small nucleic acid therapies provide an interesting new CVD treatment option and an opportunity to target disease pathways that have yet to be addressed by small molecule therapeutics. Even though ncRNA biomarker research is developing quickly, there are still a number of difficulties since preanalytical and analytical factors might affect the quality of results. Despite this apparent potential, there remain a number of challenges that must be resolved before ncRNA treatments may be used more widely in clinical settings. Developments in both carrier and modifications to RNA that would allow effective cellular absorption and intracellular stability of the molecules remain a big concern; at least until effective RNA modifications can be made, lipid-based nanocarriers now appear to be the most promising method. The prevention of endogenous activity by inhibitory miRNAs, lncRNAs, and circRNAs is currently possible by using ASO-containing LNA-MN (locked nucleic acid-modified nucleotides). LNAs against miR-15171 and AAV (adeno-associated virus) vectors expressing antisense sequences can be used to induce cardiac activity. Regeneration and cardiac activity could be enhanced by the delivery of miRNAs, lncRNAs, and circRNA mimics, which may improve the development of cardiac activity after MI. The intracardiac injection can be used to mimic miRNAs, lncRNAs, and circRNAs using various types of nanoformulations, including lipids and hydrogels. The application of cell-specific synthetic ncRNA, or ASO, may be helpful in the clinical management of CVDs. Therefore, further efforts are required to develop sensitive, specific ncRNA based on a spatiotemporal delivery approach for therapeutic interest in CVDs. There are several delivery methods used to mimic miRNAs, lncRNAs, and circRNAs, each with its own set of off-target limitations and toxicity. The selection of the material, sample isolation, detection, and processing methods, as well as normalization procedures and the impact of medications and other noncardiac disorders and phenotypes, is some of these variables. The ncRNA-based therapeutic approach is required to overcome off-target toxicity for effective clinical allocation in CVDs. The development of applications and methods related to ncRNA is still under investigation. We need to explore genome-wide analysis; next-generation sequencing and animal model studies will explore more about unknown ncRNAs. We can focus on the conserved nature of ncRNA in humans, cells, and tissue-specific regulations. Moreover, we can focus on circulating ncRNA body fluids, including serum, plasma, and urine, which can be used for diagnosis, prognosis, and treatments based on clinical signs and symptoms in patients with CVDs. CircRNA could be more effective for diagnosis and prognosis. Therefore, the biological and clinical importance of ncRNA in CVD remains unknown. Many unknown functions need to be clarified. Many undiscovered functions are still being developed. Therefore, a thorough investigation of the function of ncRNAs in CVD will lay the groundwork for novel clinical disease treatment initiatives.

## 7. Conclusions

We have reviewed significant recent advancements and clinical perspectives of ncRNA research, with special emphasis on major problems related to the conversion of preclinical and basic scientific discoveries into innovative diagnostics and therapies with clinical use. To provide accurate and repeatable results in diagnostics, it is necessary to remove the ncRNA heterogeneity based on technical and analytical factors, use reliable extraction techniques, and standardize the process. This is crucial to automate work processes and set the stage for clinical routine. Large-scale clinical trials are required to accurately evaluate the potential of ncRNAs as clinical therapeutic agents because reported results are often validated. Future clinical applications must carefully evaluate established selection criteria, appropriate clinical outcome metrics, and patient cohorts in which one pathomechanism is the single or main cause of disease development and progression. Future clinical trials will consequently likely focus on patients for whom the extra effort of ncRNA treatment techniques is likely to result in clinical success.

## Figures and Tables

**Table 1:** Treatments using non-coding RNA are being developed.

Name of the Product	Name of the Manufacturer	Functionalization	Route of Administration	Indication	Target Delivery Organ	Target mRNA or miRNA	Phase	Clinical Trial Identification Number
Vupanorsen	Ionis Pharmaceuticals, Pfizer	ASO (Antisense oligonucleotides) (GalNAc-conjugated)	Subcutaneous	Familial Chylomicronaemia Syndrome (FCS)	Liver	ANGPTL3 (angiopoietin-like 3) mRNA	Phase 2	NCT03371355
Olpasiran	Amgen	siRNA (Small interfering RNA) (GalNAc-conjugated)	Subcutaneous	Elevated Lp(a)	Liver	LPA (Lipoprotein A) mRNA	Phase 2	NCT04270760
SLN360	Silence Therapeutics	siRNA (GalNAc-conjugated)	Subcutaneous	Elevated Lp(a)	Liver	LPA mRNA	Phase 1	NCT04606602
MRG-110	miRagen, Servier	ASO (LNA)	Intradermal	Neovascularization, wound healing	Vasculature	miR-92a-3p	Phase 1	NCT03494712
ARO-ANG3	Arrowhead Pharmaceuticals	siRNA (liver targeted)	Subcutaneous	Mixed dyslipidaemia	Liver	ANGPTL3 mRNA	Phase 2	NCT04832971
MRG-110	miRagen, Servier	ASO (LNA)	Intradermal	Neovascularization, wound healing	Vasculature	miR-92a-3p	Phase 1	NCT03494712
CDR132L	Cardior	ASO (LNA)	Intravenous	Heart failure	Heart	miR-132-3p	Phase 1b	NCT04045405
elacarsen	Lonis Pharmaceuticals	ASO (PS, 2′-MOE, GalNAc-conjugated)	Subcutaneous	Elevated Lp(a)	Liver	*LPA* mRNA	Phase 3	NCT04023552
Vutrisiran	Anylam Pharmaceuticals	siRNA (GalNAc-conjugated)	Subcutaneous	TT-amyloid with cardiomyopathy	Liver	Transthyretin mRNA	Phase 3	NCT03759379
							Phase 3	NCT04153149
Teprasiran	Quark Pharmaceuticals	siRNA	Intravenous	Acute kidney injury	Kidney	p53 mRNA	Phase 2	NCT02610283
							Phase 3	NCT03510897

**Table 2:** Regulations of miRNA and their clinical importance in CVDs.

S.N.	Type of Disease	miRNA	Regulation	Importance	Reference
1	Cardiac hypertrophy (CH)	miR-208a	Up-regulation	Cardiac remodelling	[13]
2	CH	miR-19a/b	Up-regulation	Cardiac remodelling in response to angiotensin II infusion	[14]
3	CH	miR-155	Up-regulation	Cardiac remodelling	[15]
4	CH	miR-199a	Up-regulation	Maintenance of cell size in cardiomyocytes	[16]
5	CH	miR-1,	Down-regulation	Induces cardiac hypertrophy.	[17]
6	CH	miR-101	Down-regulation	Inhibit cardiac hypertrophy signalling	[18]
7	CH	miR-185	Down-regulation	Inhibit CH hypertrophy signalling	[19]
8	CH	miR-34a	Down-regulation	Regulation of Ang II-induced cardi myocyte hypertrophy	[20]
9	CH	miR-145	Down-regulation	Inhibits isoproterenol-induced cardiomyocyte hypertrophy	[21]
10	CH	miR-150	Down-regulation	Reduces the immunosuppression function of Myeloid-derived suppressor cells (MDSCs)	[22]
11	CH	miR-378	Down-regulation	Act as negative regulator for CH	[23]
12	Heart failure (HF)	miR-125b	Up-regulation	Conduction of Cardiac fibrosis (CF)	[24]
13	HF	miR-22,	Up-regulation	Regulator for cardiac remodelling	
14	HF	miR-92b	Up-regulation	Related to the left atrium diameter, left ventricular end-diastolic dimension	[25]
15	HF	miR-320a	Up-regulation	CF through activation of the IL6/STAT3 axis.	[26]
16	HF	miR-423-5p	Up-regulation	Upregulated in human failing myocardium	[27]
17	HF	miR-200b	Up-regulation	Regulation of multiple cellular pathways in HF	[28]
18	HF	miR-622	Up-regulation	Improves blood vessel growth	[29]
19	HF	miR-1228	Up-regulation	Marker for systolic HF	[30]
20	HF	miR-208b	Up-regulation	pathogenesis of DCM	[31]
21	HF	miR-499	Up-regulation	Cardiac development	[32]
22	HF	miR-223	Up-regulation	Altered in post-MI HF in humans	[33]
23	HF	miR-1254	Up-regulation	Altered in post-MI HF in humans	[34]
24	HF	miR-1306	Up-regulation	Elected to explore novel circulating markers for HF	[35]
25	HF	miR-18a	Down-regulation	CF through the Notch2 pathway.	[36]
26	HF	miR-26b	Down-regulation	Controlling critical signalling pathways, such as BMP/ SMAD1 signalling	[37]
27	HF	miR-27a	Down-regulation	Inhibiting miR-27a-3p mitigated CH phenotype induced by Ang II (Angiotensin -II)	[38]
28	HF	miR-30e	Down-regulation	The overexpression of miR-30c reduces the level of connective tissue growth	[39]
29	HF	miR-106a	Down-regulation	Notch 3 pathway in ischemic heart injury.	[40]
30	HF	miR-199a	Down-regulation	Improves contractile function	[41]
31	HF	miR-652	Down-regulation	Marker for predicting acute coronary syndrome	[42]
32	HF	miR-1	Down-regulation	Systolic HF	[43]
33	HF	miR-126	Down-regulation	Activation of the vascular endothelial growth factor (VEGM) signalling pathway in the endothelium.	[44]
34	HF	miR-423	Down-regulation	It is a circulating biomarker for heart failure.	[45]
35	Cardiac electrical and structural remodelling (CE and SR)	miR-1	Down-regulated	Increased altered conduction Increased CF	[46]
36	CE and SR	miR-26	Down-regulated	Increase inwardly rectifying channel	[47]
37	CE and SR	miR-29	Down-regulated	Increased CF	[48]
38	CE and SR	miR-30	Down-regulated	Increased CF	[49]
39	CE and SR	miR-133	Down-regulated	Increased CF	[50]
40	CE and SR	miR-328	Up-regulated	Shortened atrial action potential duration by targeting	[51]
41	CE and SR	miR-499	Up-regulated	Altered conduction by targeting	[52]
42	CE and SR	miR-21	Up-regulated	Inhibition of fibroblast proliferation	[53]
43	Acute coronary syndrome (ACS) and myocardial infarction (MI)	miR-1	Up-regulated	marker of cardiomyocyte injury	[54]
44	ACS and MI	miR-133a	Up-regulated	Development of VF (Ventricular fibrillation)	[55]
45	ACS and MI	miR-208a	Up-regulated	Regulates the cardiac stress response.	[56]
46	ACS and MI	miR-499-5p	Up-regulated	Associated with cardiac injury and also with cardio protection	[57]
47	ACS and MI	miR-126,	Down-regulated	downregulated in the region adjacent to MI areas	[58]
48	ACS and MI	miR-221/222	Down-regulated	Severity of the coronary artery lesions	[59]
49	ACS and MI	miR-29	Dysregulation	Involved in CF multiple collagens, fibrillin’s, and elastin	[60]
50	ACS and MI	miR-145	Up-regulated	Significantly upregulated in mice in response to chronic hypoxia and that genetic ablation	[61]
51	ACS and MI	miR-21	Up-regulated	Up-regulated in the hypoxia	[62]
52	ACS and MI	miR-206	Up-regulated	Normal and hypertensive mouse PASMCs.	[63]
53	ACS and MI	miR-328	Down-regulated	Regulates Hypoxic Pulmonary Hypertension	[64]
54	Pulmonary arterial hypertension (PAH)	miR-204	Down-regulated	Hypoxia related to pulmonary arterial hypertension	[65]
55	Atherosclerosis (AS)	miR-33	Dysregulation	Promising strategy to reverse autophagy dysfunction in atherosclerosis.	[66]
56	(AS)	miR-122	Up	Significantly up-regulated in patients with atherosclerotic lesion	[67]
57	(AS)	miR-126	Down-regulated		[68]
58	(AS)	miR-1	Down-regulated	Downregulation of miR-10a enhances IκB/NF-κB activation	[69]
59	(AS)	miR-221/222	Down-regulated	Suppression of PGC-1α (peroxisome proliferator-activated receptor gamma coactivator 1-alpha) in the progression of atherosclerosis	[70]
60	Congenital heart diseases (CHDs)	miR-1275, miR-27b, miR-421	Up-regulated	usually developing hearts	[13]
61	CHD	miR-122, miR-1201	Down-regulated	developing hearts	[14]
62	CHD	miR-222, miR-337-5p, miR-363, miR-424, miR-424, miR-660, miR-708, miR-421, miR-19a, miR-130b, miR-146b-5p, miR-154, miR-155, miR-181c, miR-181d and miR-192,	Up-regulated	tetralogy of Fallot	[15]
63	CHDs	miR-181a, miR-720, miR-29c and miR-940	Down-regulated	tetralogy of Fallot	[16]
64	CHD	miR-181c	Up-regulated	ventricular septal defect	[17]
	CHD	miR-1-1	Down-regulated	ventricular septal defect	[18]
65	CHD	miR-106a, miR-144, miR-451, miR-486-3p, miR-486-5p, hsa-let-7e, miR-16, miR-18a, miR-25, miR-93, and miR-505	Up-regulated	transposition of the great arteries	[19]
66	CHD	miR-873	Up-regulated	Cyanotic CHD	[20]
67	CHD	miR-182	Down-regulated	Cyanotic CHD	[21]
68	CHD	miR-498	Up-regulated	Ventricular septal defect	
69	CHD	miR-379-5p, miR-409-3p, miR-433, hsa-let-7e-5p, miR-155-5p, miR-222-3p, and miR-487b	Down-regulated	Ventricular septal defect	[22]
70	CHD	hsa-let-7b, hsa-let-7a, and miR-486	Up-regulated	Atrioventricular septal defect and atrial septal defect	[23]
71	CHD	miR-19b, miR-22, miR-29c, miR-375	Up-regulated	Atrioventricular septal defect and atrial septal defect	[22,23]
72	Myocrdial infraction (I)	miR-1		Cardiomyocyte Downstream Targets: Ncx-1; KCNJ2, GJA1; IGF-1	[55]
73	MI	miR-15	Up-regulated	Cardiomyocyte Downstream Targets: Pdk4, Sgk1	[56]
74	MI	miR-21	Down-regulated	Fibroblast, Downstream Targets Pten; Sprouty-1, collagens	[57]
75	MI	miR-24	Up-regulated	Anti-apoptosis in Cardiomyocyte, fibroblast, endothelial cell; Downstream Targets Bim; Furin; Gata2, Pak4	[58]
76	MI	miR-29	Down-regulated	Cardiomyocyte, fibroblast Downstream Targets: Mcl-1; Collagens	[59]
77	MI	miR-92a	Up-regulated	Endothelial cell Downstream Targets: Itga5	[60]
78	MI	miR-101	Down-regulated	Cardiac remodelling Downstream Targets: Collagens	[61]
79	MI	miR-126	Down-regulated	Protects against myocardial ischemia-reperfusion injury	[62]

**Table 3:** Regulations of lncRNA in cardiovascular disorders and their clinical significance.

	Type of Disease	lncRNA	Regulations	Importance	References
1	Myocardial infraction (MI)	aHIF	Regulations of the angiogenesis process and a biomarker	Inhibits the autophagy of cardiac cells during MI	[102]
2	MI	ANRIL	Regulates myocardial cell apoptosis in AMI	Protection of cardiomyocytes	[103]
3	MI	APF	APF lncRNA regulates autophagy	Acting as a sponge for miRNA- 188-3p.	[102]
4	MI	CARL	Regulates mitochondrial fission and apoptosis	Acting as a sponge for miRNA-539.	[102]
5	MI	CDR1AS	Inhibiting the autophagy of cardiac cells during *MI*	Biomarker.	[102]
6	MI	FTX	Regulates cardiomyocytes	Act as a sponge for miRNA- 29b-1-5.	[104]
7	MI	GAS5	Regulates the protection of cardiomyocytes against hypoxic injury	Act as a sponge for miRNA-142; improves apoptosis by negatively regulating sema3a.	[102]
8	MI	H19	Regulates autophagy	Induction of cardiac remodeling, autophagy, and biomarker.	[102]
9	MI	HOTAIR	Regulates cardioprotective	Act as a sponge for miRNA-1 and as a biomarker.	[105]
10	MI	KCNQ1OT1	Down-regulation of lncRNA KCNQ1OT1 protects against myocardial ischemia/reperfusion injury	Biomarker for left ventricular dysfunction.	[106]
11	MI	LIPCAR	Down-regulated	Biomarker for cardiac remodelling.	[107]
12	MI	Lnc-Ang362	Upregulation	Promotion of CF	[108]
	MI	MALAT1	Down-regulation	Regulation of cardiomyocytes apoptosis and autophagy through miRNA- 558; and biomarker.	[109]
13	MI	MDRL	Regulates mitochondrial fission	Reduction of mitochondrial fission and apoptosis acting as a sponge for miRNA-361.	[110]
14	MI	MEG3	Regulates cardiomyocytes	Regulation of cardiomyocytes apoptosis.	[111]
15	MI	MHRT	Regulates cardiomyocytes	Regulation of cardiomyocytes apoptosis and biomarker.	[112]
16	MI	MIAT	Regulates CF	Regulation of cardiac hypertrophy and fibrosis acting as a sponge for miRNA-150 and -93.	[102]
17	MI	Mirt1/2	Regulates cardiomyocytes	Regulation of cardiac remodelling.	[102]
18	MI	n379519	Regulates CF	Promotion of cardiac fibrosis through miRNA-30.	[113]
19	MI	NONRATT021972	Regulates cardiomyocytes	Promotion of cardiac function.	[114]
20	MI	NRF	Regulates cardiomyocyte necrosis.	Regulation of cardiomyocyte necrosis.	[115]
21	MI	NRON	Up-regulated	Wisper in cardiac fibroblast; Biomarker	[102]
22	MI	PCFL	Up-regulated	Promotion of cardiac fibrosis through miRNA-378.	[102]
23	MI	TTTY15	Up-regulated	Induction of cardiomyocyte injury by hypoxia targeting miRNA-455.	[102]
24	MI	UCA1	Regulates cardiomyocytes	Regulated ischemia and hypoxia of cardiomyocytes; Biomarker.	[115]
25	MI	UIHTC	Regulates cardiomyocytes against MI	Promotion of mitochondrial function.	[102]
26	MI	Wisper	Regulates cardiac fibroblast	*MI*-induced fibrosis and cardiac dysfunction	[116]
27	MI	ZFAS1	Regulates cardiomyocyte	Induction of cardiomyocyte apoptosis, cardiac contractility reduction, and biomarker.	[117]
28	Coronary heart disease	aHIF	Up-regulated	Biomarker.	[118]
29	Coronary heart disease	ANRIL	Down-regulates	Diagnostic and prognostic indicator for *CHD*	[118]
30	Coronary heart disease	APOA1-AS	Up-regulations increase the risk of CHD	Biomarker.	[119]
31	Coronary heart disease	AWPPH	Regulates apoptosis.	Promotion of ECs apoptosis.	[120]
32	Coronary heart disease	BACE1-AS	dysregulation	dysregulation of the BACE1/BACE1-AS/Aβ axis is associated with HF.	[121]
33	Coronary heart disease	BANCR	Differentially expressed	Promotion of VSMCs proliferation and migration.	[122]
34	Coronary heart disease	CHROME	Up-regulated	Regulation of cellular cholesterol homeostasis	[123]
35	Coronary heart disease	CoroMarker	Differentially expressed	novel biomarker for the diagnosis	[124]
36	Coronary heart disease	EGOT	Differentially expressed	Biomarker.	[125]
37	Coronary heart disease	H19	Differentially expressed	Biomarker.	[126]
38	Coronary heart disease	HOTTIP	Up-regulates	Promotes ECs proliferation and migration	[127]
39	Coronary heart disease	HRCR	Regulates hypertrophic Ca^2+^ signaling pathway	Regulation of cardiomyocytes apoptosis and proliferation.	[128]
40	Coronary heart disease	LIPCAR	Differentially expressed	Biomarker.	[107]
41	Coronary heart disease	lincRNA-p21	Regulates cardiac remodelling and heart failure	Regulation of cardiomyocytes apoptosis and proliferation.	[129]
42	Coronary heart disease	LINC00968	Up-regulated	Promotion of ECs proliferation and migration acting as a sponge for miRNA-9	[130]
43	Coronary heart disease	MALAT1	Differentially expressed	Biomarker.	[131]
44	Coronary heart disease	MIAT	Differentially expressed	Biomarker	[132]
45	Coronary heart disease	NEXN-AS1	Differentially expressed	Mitigation of atherosclerosis.	[133]
46	Coronary heart disease	SMILR	Differentially expressed	Biomarker.	[134]
47	Arterial Hypertension	AK098656	Up-regulated	Regulation of arteries of resistance and a biomarker	[135]
48	Arterial Hypertension	ANRIL	Regulates endothelial cell activities	Increase of susceptibility to higher systolic blood pressure conferred by polymorphisms.	[136]
49	Arterial Hypertension	GAS5	Regulates ECs and VSMCs function	Regulation of ECs and VSMCs function acting as endogenous RNA competing of miRNA-21; and a biomarker. GAS5 Targets miR-194-3p. miR-194-3	[137]
50	Arterial Hypertension	Giver	Regulates VSMCs dysfunction.	Promotion of VSMCs dysfunction.	[138]
51	Arterial Hypertension	Lnc-Ang362	Regulates VSMCs	Regulation of VSMCs proliferation through miRNA-221 and -222.	[139]
52	Arterial Hypertension	NR_027032	Differentially expressed	Biomarker.	[140]
53	Arterial Hypertension	NR_034083	Differentially expressed	Biomarker.	[141]
54	Arterial Hypertension	NR_104181	Differentially expressed	Biomarker.	[140]
55	Heart failure	ANRIL	Differentially expressed	Biomarker.	[142]
56	Heart failure	BACE1-AS	Regulates apoptosis.	Promotion of ECs apoptosis.	[143]
57	Heart failure	Chaer	Dysregulation	Induction of Pathological cardiac remodelling.	[144]
58	Heart failure	Chast	Down-regulates	Induction of Pathological cardiac remodelling.	[145]
59	Heart failure	CHRF	Up-regulated	Endogenous sponge to miRNA-489 activity.	[146]
60	Heart failure	HEAT2	Up-regulated	Biomarker.	[147]
61	Heart failure	HOTAIR	Up-regulated	LncRNA HOTAIR may function as a miR-19-sponge to modulate PTEN levels Biomarker.	[148]
62	Heart failure	LIPCAR	Up-regulated	Biomarker.	[149]
63	Heart failure	lincRNA-ROR	Regulates CH	Regulation of cardiac hypertrophy acting as a sponge for miRNA-133.	[150]
64	Heart failure	LOC285194	Up-regulated	overexpression suppressed MKN45 and HGC-27 cell proliferation and promoted cell apoptosis; Biomarker.	[148]
65	Heart failure	MEG3	Regulates CF	Regulation of cardiac fibrosis and diastolic dysfunction	[151]
66	Heart failure	MHRT	Regulates of chromatin re-modellers	Regulation of chromatin remodels and biomarker.	[152]
67	Heart failure	MIAT	Regulates CH	Regulation of cardiac hypertrophy acting as a sponge for miRNA-150.	[153]
68	Heart failure	NRON	Upregulated	Biomarker.	[154]
69	Heart failure	RNY5	Dysregulation	Biomarker.	[155]
70	Heart failure	SOX2-OT	Dysregulation	Biomarker.	[156]
71	Heart failure	SRA1	Dysregulation	Biomarker.	[157]

**Table 4:** Regulations of circRNAs and their clinical importance in CVDs.

S.N.	Type of Disease	CircRNA	Regulation	Importance	References
1	Cardiac Hypertrophy (CH)	CircHRCR	Down-regulated attenuates cardiac hypertrophy	Down-regulated attenuates CH	[175]
2	CH	CircSLc8a1	Up-regulated	Knockdown reduces CH caused by pressure overload	[176]
3	CH	Circ_000203	Up-regulated aggravates cardiac hypertrophy	Increased CH	[177]
4	Myocardial Infarction (MI)	CircNfix	Down-regulated	Promotes cardiomyocyte proliferation and angiogenesis	[178]
5	MI	CircFndc3b	Down-regulated	Overexpression increases ECF (endothelial cell function)	[179]
6	MI	CircTtc3	Up-regulated	Inhibits apoptosis of cardiomyocytes	[180]
7	MI	Circ-CDR1as	Up-regulated	Promotes cell apoptosis	[181]
8	MI	CircMFACR	Up-regulated	Induces autophagy and cell death in cardiomyocytes	[182]
9	Myocardial fibrosis (MF)	CircNFIB	Down-regulated	Increases proliferation and differentiation of myocardial fibroblasts (MF)	[183]
10	MF	CircHIPK3	Up-regulated	Overexpression attenuates the proliferation and migration of CF	[184]
11	MF	Circ_Las1l	Down-regulated	Regulates proliferation and migration, and apoptosis	[185]
12	MF	CircRNA-010567	Up-regulated	Silencing inhibits the CF	[186]
13	Atherosclerosis (AS)	CircANRIL	Down-regulated monocytic	Regulates increase in a apoptosis and decrease in proliferation	[187]
14	AS	Circ_0003204	Up-regulated	Ectopic expression inhibits proliferation, migration, and endothelial cells	[172]
15	AS	CircWDR77	Up-regulated	Silencing inhibits VSMC proliferation and migration	[181]
16	AS	Circ_0003575	Unchanged	Increase proliferation and angiogenesis	[186]
17	Cardiac senescence	CircAmotl1	Down-regulated during aging	Ectopic expression induces primary cardiomyocyte proliferation	[188]
18	Cardiac senescence	CircFOXO3	Up-regulated	Ectopic expression induces senescence	[189]
19	Coronary heart disease (CHD)	CircZNF60	Up-regulated	Silencing increases, proliferation, and migration	[190]
20	CHD	CircLrp6	Unchanged	Silencing prevents intimal	[168]
21	CHD	CircNrg1	Down-regulated	Knockdown inhibits the apoptosis	[191]
22	CHD	CircTCF25	Down-regulated	Down-regulated expression in coronary heart disease	[192]

**Table 5:** The main non-coding RNA treatments for cardiovascular applications are in development.

Target mRNA or miRNA	Type (Modifications)	Route of Administration	Target mRNA or miRNA	Product (Developer/Manufacturer)	Indication	Phase	Latest Clinical Studies
LPA mRNA	Antisense oligonucleotide (ASO) (PS, 2′-MOE, GalNAc-conjugated	Subcutaneous	LPA mRNA	elacarsen (Ionis Pharmaceuticals)	Elevated Lp(a)	Phase 3	NCT0402355
LPA mRNA	siRNA (GalNAc-conjugated)	Subcutaneous	LPA mRNA	Olpasiran (Amgen)	Elevated Lp(a)	Phase 2	NCT04270760
*LPA* mRNA	siRNA (GalNAc-conjugated)	Subcutaneou	*LPA* mRNA	SLN360 (Silence Therapeutics)	Elevated Lp(a)	Phase 1	NCT046066
ANGPTL3 mRNA	ASO (GalNAc-conjugated)	Subcutaneous	ANGPTL3 mRNA	Vupanorsen (Ionis Pharmaceuticals, Pfizer)	Hypertriglyceridemia; Familial Chylomicronemia Syndrome (FCS)	Phase 2	NCT0337135 NCT04516291
ANGPTL3 mRNA	siRNA (liver targeted)	Subcutaneous	ANGPTL3 mRNA	ARO-ANG3 (Arrowhead Pharmaceuticals)	Mixed dyslipidaemia	Phase 2	NCT04832971
miR-92a-3p	ASO (LNA)	Intradermal	miR-92a-3p	MRG-110 (S95010) (miRagen, Servier)	Neovascularization, wound healing	Phase 1	NCT03494712; NCT03603431
miR-132-3p	ASO (LNA)	Intravenous	miR-132-3p	CDR132L (Cardior)	Heart failure	Phase 1	NCT0404540
p53 mRNA	siRNA (short interfering RNA)	Intravenous	p53 mRNA	Teprasiran (Quark Pharmaceuticals)	Acute kidney injury	Phase 3	NCT03510897
Transthyretin mRNA	siRNA (GalNAc-conjugated)	Subcutaneous	Transthyretin mRNA	Vutrisiran (Anylam Pharmaceuticals)	HTT-amyloid with polyneuropathy; TT-amyloid with cardiomyopathy	Phase 3	NCT04153149

## Data Availability

Not applicable.

## References

[1] Roth G.A., Mensah G.A., Johnson C.O., Addolorato G., Ammirati E., Baddour L.M., Barengo N.C., Beaton A.Z., Benjamin E.J., Benziger C.P. (2020). Global Burden of Cardiovascular Diseases and Risk Factors, 1990–2019. J. Am. Coll. Cardiol..

[2] Sreeniwas Kumar A., Sinha N. (2020). Cardiovascular Disease in India: A 360 Degree Overview. Med. J. Armed Forces India.

[3] Cuadrado-Godia E., Ois A., Roquer J. (2010). Heart Failure in Acute Ischemic Stroke. CCR.

[4] Frangogiannis N.G. (2021). Cardiac Fibrosis. Cardiovasc. Res..

[5] Schwalm J.D., McKee M., Huffman M.D., Yusuf S. (2016). Resource Effective Strategies to Prevent and Treat Cardiovascular Disease. Circulation.

[6] Sallam T., Sandhu J., Tontonoz P. (2018). Long Noncoding RNA Discovery in Cardiovascular Disease: Decoding Form to Function. Circ. Res..

[7] Zhang C., Han B., Xu T., Li D. (2020). The Biological Function and Potential Mechanism of Long Non-coding RNAs in Cardiovascular Disease. J. Cell. Mol. Med..

[8] Correia C.C.M., Rodrigues L.F., de Avila Pelozin B.R., Oliveira E.M., Fernandes T. (2021). Long Non-Coding RNAs in Cardiovascular Diseases: Potential Function as Biomarkers and Therapeutic Targets of Exercise Training. ncRNA.

[9] Poller W., Dimmeler S., Heymans S., Zeller T., Haas J., Karakas M., Leistner D.-M., Jakob P., Nakagawa S., Blankenberg S. (2018). Non-Coding RNAs in Cardiovascular Diseases: Diagnostic and Therapeutic Perspectives. Eur. Heart J..

[10] Lu P., Ding F., Xiang Y.K., Hao L., Zhao M. (2022). Noncoding RNAs in Cardiac Hypertrophy and Heart Failure. Cells.

[11] Huang C.-K., Kafert-Kasting S., Thum T. (2020). Preclinical and Clinical Development of Noncoding RNA Therapeutics for Cardiovascular Disease. Circ. Res..

[12] Marinescu M.-C., Lazar A.-L., Marta M.M., Cozma A., Catana C.-S. (2022). Non-Coding RNAs: Prevention, Diagnosis, and Treatment in Myocardial Ischemia–Reperfusion Injury. Int. J. Mol. Sci..

[13] Huang X.-H., Li J.-L., Li X.-Y., Wang S.-X., Jiao Z.-H., Li S.-Q., Liu J., Ding J. (2021). MiR-208a in Cardiac Hypertrophy and Remodeling. Front. Cardiovasc. Med..

[14] Liu K., Hao Q., Wei J., Li G.-H., Wu Y., Zhao Y.-F. (2018). MicroRNA-19a/b-3p Protect the Heart from Hypertension-Induced Pathological Cardiac Hypertrophy through PDE5A. J. Hypertens..

[15] Seok H.Y., Chen J., Kataoka M., Huang Z.-P., Ding J., Yan J., Hu X., Wang D.-Z. (2014). Loss of MicroRNA-155 Protects the Heart From Pathological Cardiac Hypertrophy. Circ. Res..

[16] Yan M., Yang S., Meng F., Zhao Z., Tian Z., Yang P. (2018). MicroRNA 199a-5p Induces Apoptosis by Targeting JunB. Sci. Rep..

[17] Wehbe N., Nasser S., Pintus G., Badran A., Eid A., Baydoun E. (2019). MicroRNAs in Cardiac Hypertrophy. Int. J. Mol. Sci..

[18] Wei L., Yuan M., Zhou R., Bai Q., Zhang W., Zhang M., Huang Y., Shi L. (2015). MicroRNA-101 Inhibits Rat Cardiac Hypertrophy by Targeting Rab1a. J. Cardiovasc. Pharmacol..

[19] Kim J.O., Song D.W., Kwon E.J., Hong S.-E., Song H.K., Min C.K., Kim D.H. (2015). MiR-185 Plays an Anti-Hypertrophic Role in the Heart via Multiple Targets in the Calcium-Signaling Pathways. PLoS ONE.

[20] Huang J., Sun W., Huang H., Ye J., Pan W., Zhong Y., Cheng C., You X., Liu B., Xiong L. (2014). MiR-34a Modulates Angiotensin II-Induced Myocardial Hypertrophy by Direct Inhibition of ATG9A Expression and Autophagic Activity. PLoS ONE.

[21] Li R., Yan G., Zhang Q., Jiang Y., Sun H., Hu Y., Sun J., Xu B. (2013). MiR-145 Inhibits Isoproterenol-induced Cardiomyocyte Hypertrophy by Targeting the Expression and Localization of GATA6. FEBS Lett..

[22] Liu W., Liu Y., Zhang Y., Zhu X., Zhang R., Guan L., Tang Q., Jiang H., Huang C., Huang H. (2015). MicroRNA-150 Protects Against Pressure Overload-Induced Cardiac Hypertrophy: M ICRO RNA-150 M ODULATES C ARDIAC H YPERTROPHY. J. Cell. Biochem..

[23] Ganesan J., Ramanujam D., Sassi Y., Ahles A., Jentzsch C., Werfel S., Leierseder S., Loyer X., Giacca M., Zentilin L. (2013). MiR-378 Controls Cardiac Hypertrophy by Combined Repression of Mitogen-Activated Protein Kinase Pathway Factors. Circulation.

[24] Zhang B., Mao S., Liu X., Li S., Zhou H., Gu Y., Liu W., Fu L., Liao C., Wang P. (2021). MiR-125b Inhibits Cardiomyocyte Apoptosis by Targeting BAK1 in Heart Failure. Mol. Med..

[25] Huang Z.-P., Wang D.-Z. (2014). MiR-22 in Cardiac Remodeling and Disease. Trends Cardiovasc. Med..

[26] Li F., Li S.-S., Chen H., Zhao J.-Z., Hao J., Liu J.-M., Zu X.-G., Cui W. (2021). MiR-320 Accelerates Chronic Heart Failure with Cardiac Fibrosis through Activation of the IL6/STAT3 Axis. Aging.

[27] Tijsen A.J., Creemers E.E., Moerland P.D., de Windt L.J., van der Wal A.C., Kok W.E., Pinto Y.M. (2010). MiR423-5p As a Circulating Biomarker for Heart Failure. Circ. Res..

[28] Zhang F., Cheng N., Du J., Zhang H., Zhang C. (2021). MicroRNA-200b-3p Promotes Endothelial Cell Apoptosis by Targeting HDAC4 in Atherosclerosis. BMC Cardiovasc. Disord.

[29] Shen N.-N., Wang J.-L., Fu Y. (2022). The MicroRNA Expression Profiling in Heart Failure: A Systematic Review and Meta-Analysis. Front. Cardiovasc. Med..

[30] Peterlin A., Počivavšek K., Petrovič D., Peterlin B. (2020). The Role of MicroRNAs in Heart Failure: A Systematic Review. Front. Cardiovasc. Med..

[31] Zhao X., Wang Y., Sun X. (2020). The Functions of MicroRNA-208 in the Heart. Diabetes Res. Clin. Pract..

[32] Khanaghaei M., Tourkianvalashani F., Hekmatimoghaddam S., Ghasemi N., Rahaie M., Khorramshahi V., Sheikhpour A., Heydari Z., Pourrajab F. (2016). Circulating MiR-126 and MiR-499 Reflect Progression of Cardiovascular Disease; Correlations with Uric Acid and Ejection Fraction. Heart Int..

[33] Zhang M.-W., Shen Y.-J., Shi J., Yu J.-G. (2021). MiR-223-3p in Cardiovascular Diseases: A Biomarker and Potential Therapeutic Target. Front. Cardiovasc. Med..

[34] De Gonzalo-Calvo D., Cediel G., Bär C., Núñez J., Revuelta-Lopez E., Gavara J., Ríos-Navarro C., Llorente-Cortes V., Bodí V., Thum T. (2018). Circulating MiR-1254 Predicts Ventricular Remodeling in Patients with ST-Segment-Elevation Myocardial Infarction: A Cardiovascular Magnetic Resonance Study. Sci. Rep..

[35] Chen X., Li C., Li J., Sheng L., Liu X. (2019). Upregulation of MiR-1306-5p Decreases Cerebral Ischemia/Reperfusion Injury in Vitro by Targeting BIK. Biosci. Biotechnol. Biochem..

[36] Yuan L., Tang C., Li D., Yang Z. (2018). MicroRNA-18a Expression in Female Coronary Heart Disease and Regulatory Mechanism on Endothelial Cell by Targeting Estrogen Receptor. J. Cardiovasc. Pharmacol..

[37] Icli B., Dorbala P., Feinberg M.W. (2014). An Emerging Role for the MiR-26 Family in Cardiovascular Disease. Trends Cardiovasc. Med..

[38] Tian C., Hu G., Gao L., Hackfort B.T., Zucker I.H. (2020). Extracellular Vesicular MicroRNA-27a* Contributes to Cardiac Hypertrophy in Chronic Heart Failure. J. Mol. Cell. Cardiol..

[39] Yang J., Yang X.-S., Fan S.-W., Zhao X.-Y., Li C., Zhao Z.-Y., Pei H.-J., Qiu L., Zhuang X., Yang C.-H. (2021). Prognostic Value of MicroRNAs in Heart Failure: A Meta-Analysis. Medicine.

[40] Guan X., Wang L., Liu Z., Guo X., Jiang Y., Lu Y., Peng Y., Liu T., Yang B., Shan H. (2016). MiR-106a Promotes Cardiac Hypertrophy by Targeting Mitofusin 2. J. Mol. Cell. Cardiol..

[41] Gabisonia K., Prosdocimo G., Aquaro G.D., Carlucci L., Zentilin L., Secco I., Ali H., Braga L., Gorgodze N., Bernini F. (2019). MicroRNA Therapy Stimulates Uncontrolled Cardiac Repair after Myocardial Infarction in Pigs. Nature.

[42] Chi X., Jiang Y., Chen Y., Lv L., Chen J., Yang F., Zhang X., Pan F., Cai Q. (2021). Upregulation of MicroRNA MiR-652-3p Is a Prognostic Risk Factor for Hepatocellular Carcinoma and Regulates Cell Proliferation, Migration, and Invasion. Bioengineered.

[43] Kura B., Kalocayova B., Devaux Y., Bartekova M. (2020). Potential Clinical Implications of MiR-1 and MiR-21 in Heart Disease and Cardioprotection. Int. J. Mol. Sci..

[44] Wang X., Lian Y., Wen X., Guo J., Wang Z., Jiang S., Hu Y. (2017). Expression of MiR-126 and Its Potential Function in Coronary Artery Disease. Afr. Health Sci..

[45] Rizzacasa B., Morini E., Mango R., Vancheri C., Budassi S., Massaro G., Maletta S., Macrini M., D’Annibale S., Romeo F. (2019). MiR-423 Is Differentially Expressed in Patients with Stable and Unstable Coronary Artery Disease: A Pilot Study. PLoS ONE.

[46] Fathi M., Gharakhanlou R., Rezaei R. (2020). The Changes Of Heart MiR-1 And MiR-133 Expressions Following Physiological Hypertrophy Due To Endurance Training. Cell J..

[47] Luo X., Pan Z., Shan H., Xiao J., Sun X., Wang N., Lin H., Xiao L., Maguy A., Qi X.-Y. (2013). MicroRNA-26 Governs Profibrillatory Inward-Rectifier Potassium Current Changes in Atrial Fibrillation. J. Clin. Investig..

[48] Sassi Y., Avramopoulos P., Ramanujam D., Grüter L., Werfel S., Giosele S., Brunner A.-D., Esfandyari D., Papadopoulou A.S., De Strooper B. (2017). Cardiac Myocyte MiR-29 Promotes Pathological Remodeling of the Heart by Activating Wnt Signaling. Nat. Commun..

[49] Li J., Salvador A.M., Li G., Valkov N., Ziegler O., Yeri A., Yang Xiao C., Meechoovet B., Alsop E., Rodosthenous R.S. (2021). Mir-30d Regulates Cardiac Remodeling by Intracellular and Paracrine Signaling. Circ. Res..

[50] Li N., Zhou H., Tang Q. (2018). MiR-133: A Suppressor of Cardiac Remodeling?. Front. Pharmacol..

[51] Huang H., Chen H., Liang X., Chen X., Chen X., Chen C. (2022). Upregulated MiR-328-3p and Its High Risk in Atrial Fibrillation: A Systematic Review and Meta-Analysis with Meta-Regression. Medicine.

[52] Ling T.-Y., Wang X.-L., Chai Q., Lau T.-W., Koestler C.M., Park S.J., Daly R.C., Greason K.L., Jen J., Wu L.-Q. (2013). Regulation of the SK3 Channel by MicroRNA-499—Potential Role in Atrial Fibrillation. Heart Rhythm..

[53] Cardin S., Guasch E., Luo X., Naud P., Le Quang K., Shi Y., Tardif J.-C., Comtois P., Nattel S. (2012). Role for MicroRNA-21 in Atrial Profibrillatory Fibrotic Remodeling Associated With Experimental Postinfarction Heart Failure. Circ. Arrhythmia Electrophysiol..

[54] Girmatsion Z., Biliczki P., Bonauer A., Wimmer-Greinecker G., Scherer M., Moritz A., Bukowska A., Goette A., Nattel S., Hohnloser S.H. (2009). Changes in MicroRNA-1 Expression and IK1 up-Regulation in Human Atrial Fibrillation. Heart Rhythm..

[55] Wexler Y., Nussinovitch U. (2020). The Diagnostic Value of Mir-133a in ST Elevation and Non-ST Elevation Myocardial Infarction: A Meta-Analysis. Cells.

[56] Wang J., Xu L., Tian L., Sun Q. (2021). Circulating MicroRNA-208 Family as Early Diagnostic Biomarkers for Acute Myocardial Infarction: A Meta-Analysis. Medicine.

[57] Hoekstra M. (2016). MicroRNA-499-5p: A Therapeutic Target in the Context of Cardiovascular Disease. Ann. Transl. Med..

[58] Ling H., Guo Z., Shi Y., Zhang L., Song C. (2020). Serum Exosomal MicroRNA-21, MicroRNA-126, and PTEN Are Novel Biomarkers for Diagnosis of Acute Coronary Syndrome. Front. Physiol..

[59] Yu X., Xu J., Song M., Zhang L., Li Y., Han L., Tang M., Zhang W., Zhong M., Wang Z. (2022). Associations of Circulating MicroRNA-221 and 222 With the Severity of Coronary Artery Lesions in Acute Coronary Syndrome Patients. Angiology.

[60] Rusu-Nastase E.G., Lupan A.-M., Marinescu C.I., Neculachi C.A., Preda M.B., Burlacu A. (2022). MiR-29a Increase in Aging May Function as a Compensatory Mechanism Against Cardiac Fibrosis Through SERPINH1 Downregulation. Front. Cardiovasc. Med..

[61] Caruso P., Dempsie Y., Stevens H.C., McDonald R.A., Long L., Lu R., White K., Mair K.M., McClure J.D., Southwood M. (2012). A Role for MiR-145 in Pulmonary Arterial Hypertension: Evidence From Mouse Models and Patient Samples. Circ. Res..

[62] Parikh V.N., Jin R.C., Rabello S., Gulbahce N., White K., Hale A., Cottrill K.A., Shaik R.S., Waxman A.B., Zhang Y.-Y. (2012). MicroRNA-21 Integrates Pathogenic Signaling to Control Pulmonary Hypertension: Results of a Network Bioinformatics Approach. Circulation.

[63] Jalali S., Ramanathan G.K., Parthasarathy P.T., Aljubran S., Galam L., Yunus A., Garcia S., Cox R.R., Lockey R.F., Kolliputi N. (2012). Mir-206 Regulates Pulmonary Artery Smooth Muscle Cell Proliferation and Differentiation. PLoS ONE.

[64] Guo L., Qiu Z., Wei L., Yu X., Gao X., Jiang S., Tian H., Jiang C., Zhu D. (2012). The MicroRNA-328 Regulates Hypoxic Pulmonary Hypertension by Targeting at Insulin Growth Factor 1 Receptor and L-Type Calcium Channel-A1C. Hypertension.

[65] Courboulin A., Paulin R., Giguère N.J., Saksouk N., Perreault T., Meloche J., Paquet E.R., Biardel S., Provencher S., Côté J. (2011). Role for MiR-204 in Human Pulmonary Arterial Hypertension. J. Exp. Med..

[66] Ouimet M., Ediriweera H., Afonso M.S., Ramkhelawon B., Singaravelu R., Liao X., Bandler R.C., Rahman K., Fisher E.A., Rayner K.J. (2017). MicroRNA-33 Regulates Macrophage Autophagy in Atherosclerosis. ATVB.

[67] Wu X., Du X., Yang Y., Liu X., Liu X., Zhang N., Li Y., Jiang X., Jiang Y., Yang Z. (2021). Inhibition of MiR-122 Reduced Atherosclerotic Lesion Formation by Regulating NPAS3-Mediated Endothelial to Mesenchymal Transition. Life Sci..

[68] Boon R.A., Dimmeler S. (2014). MicroRNA-126 in Atherosclerosis. ATVB.

[69] Šatrauskienė A., Navickas R., Laucevičius A., Krilavičius T., Užupytė R., Zdanytė M., Ryliškytė L., Jucevičienė A., Holvoet P. (2021). Mir-1, MiR-122, MiR-132, and MiR-133 Are Related to Subclinical Aortic Atherosclerosis Associated with Metabolic Syndrome. Int. J. Environ. Res. Public Health.

[70] Song J., Ouyang Y., Che J., Li X., Zhao Y., Yang K., Zhao X., Chen Y., Fan C., Yuan W. (2017). Potential Value of MiR-221/222 as Diagnostic, Prognostic, and Therapeutic Biomarkers for Diseases. Front. Immunol..

[71] Lee R.C., Feinbaum R.L., Ambros V. (1993). The C. Elegans Heterochronic Gene Lin-4 Encodes Small RNAs with Antisense Complementarity to Lin-14. Cell.

[72] Lee Y., Kim M., Han J., Yeom K.-H., Lee S., Baek S.H., Kim V.N. (2004). MicroRNA Genes Are Transcribed by RNA Polymerase II. EMBO J..

[73] Carthew R.W., Sontheimer E.J. (2009). Origins and Mechanisms of MiRNAs and SiRNAs. Cell.

[74] Zhu L., Li N., Sun L., Zheng D., Shao G. (2021). Non-Coding RNAs: The Key Detectors and Regulators in Cardiovascular Disease. Genomics.

[75] Saheera S., Krishnamurthy P. (2020). Cardiovascular Changes Associated with Hypertensive Heart Disease and Aging. Cell Transpl..

[76] Vavassori C., Cipriani E., Colombo G.I. (2022). Circulating MicroRNAs as Novel Biomarkers in Risk Assessment and Prognosis of Coronary Artery Disease. Eur. Cardiol..

[77] Knezevic I., Patel A., Sundaresan N.R., Gupta M.P., Solaro R.J., Nagalingam R.S., Gupta M. (2012). A Novel Cardiomyocyte-Enriched MicroRNA, MiR-378, Targets Insulin-like Growth Factor 1 Receptor. J. Biol. Chem..

[78] Gozuacik D., Akkoc Y., Ozturk D.G., Kocak M. (2017). Autophagy-Regulating MicroRNAs and Cancer. Front. Oncol..

[79] Ikeda S., He A., Kong S.W., Lu J., Bejar R., Bodyak N., Lee K.-H., Ma Q., Kang P.M., Golub T.R. (2009). MicroRNA-1 Negatively Regulates Expression of the Hypertrophy-Associated Calmodulin and Mef2a Genes. Mol. Cell Biol..

[80] Pfeffer M.A., Shah A.M., Borlaug B.A. (2019). Heart Failure With Preserved Ejection Fraction In Perspective. Circ. Res..

[81] Wong L., Wang J., Liew O., Richards A., Chen Y.-T. (2016). MicroRNA and Heart Failure. Int. J. Mol. Sci..

[82] Schulte C. (2015). Diagnostic and Prognostic Value of Circulating MicroRNAs in Heart Failure with Preserved and Reduced Ejection Fraction. WJC.

[83] Iwasaki Y., Nishida K., Kato T., Nattel S. (2011). Atrial Fibrillation Pathophysiology: Implications for Management. Circulation.

[84] Ultimo S., Zauli G., Martelli A.M., Vitale M., McCubrey J.A., Capitani S., Neri L.M. (2018). Cardiovascular Disease-Related MiRNAs Expression: Potential Role as Biomarkers and Effects of Training Exercise. Oncotarget.

[85] Osbourne A., Calway T., Broman M., McSharry S., Earley J., Kim G.H. (2014). Downregulation of Connexin43 by MicroRNA-130a in Cardiomyocytes Results in Cardiac Arrhythmias. J. Mol. Cell. Cardiol..

[86] Thygesen K., Alpert J.S., Jaffe A.S., Chaitman B.R., Bax J.J., Morrow D.A., White H.D. (2018). The Executive Group on behalf of the Joint European Society of Cardiology (ESC)/American College of Cardiology (ACC)/American Heart Association (AHA)/World Heart Federation (WHF) Task Force for the Universal Definition of Myocardial Infarction Fourth Universal Definition of Myocardial Infarction (2018). Circulation.

[87] Sayed A.S.M., Xia K., Yang T.-L., Peng J. (2013). Circulating MicroRNAs: A Potential Role in Diagnosis and Prognosis of Acute Myocardial Infarction. Dis. Markers.

[88] Zhou S., Jin J., Wang J., Zhang Z., Freedman J.H., Zheng Y., Cai L. (2018). MiRNAS in Cardiovascular Diseases: Potential Biomarkers, Therapeutic Targets and Challenges. Acta Pharm. Sin..

[89] Halushka P.V., Goodwin A.J., Halushka M.K. (2019). Opportunities for MicroRNAs in the Crowded Field of Cardiovascular Biomarkers. Annu. Rev. Pathol. Mech. Dis..

[90] Churov A., Summerhill V., Grechko A., Orekhova V., Orekhov A. (2019). MicroRNAs as Potential Biomarkers in Atherosclerosis. Int. J. Mol. Sci..

[91] Ali Sheikh M.S., Alduraywish A., Almaeen A., Alruwali M., Alruwaili R., Alomair B.M., Salma U., Hedeab G.M., Bugti N., A.M.Abdulhabeeb I. (2021). Therapeutic Value of MiRNAs in Coronary Artery Disease. Oxidative Med. Cell. Longev..

[92] Andreou I., Sun X., Stone P.H., Edelman E.R., Feinberg M.W. (2015). MiRNAs in Atherosclerotic Plaque Initiation, Progression, and Rupture. Trends Mol. Med..

[93] Uray K., Major E., Lontay B. (2020). MicroRNA Regulatory Pathways in the Control of the Actin–Myosin Cytoskeleton. Cells.

[94] Nappi F., Iervolino A., Avtaar Singh S.S., Chello M. (2021). MicroRNAs in Valvular Heart Diseases: Biological Regulators, Prognostic Markers and Therapeutical Targets. Int. J. Mol. Sci..

[95] Bielska A., Niemira M., Kretowski A. (2021). Recent Highlights of Research on MiRNAs as Early Potential Biomarkers for Cardiovascular Complications of Type 2 Diabetes Mellitus. Int. J. Mol. Sci..

[96] Fang Y., Xu Y., Wang R., Hu L., Guo D., Xue F., Guo W., Zhang D., Hu J., Li Y. (2020). Recent Advances on the Roles of LncRNAs in Cardiovascular Disease. J. Cell. Mol. Med..

[97] Bär C., Chatterjee S., Thum T. (2016). Long Noncoding RNAs in Cardiovascular Pathology, Diagnosis, and Therapy. Circulation.

[98] Uchida S., Dimmeler S. (2015). Long Noncoding RNAs in Cardiovascular Diseases. Circ. Res..

[99] Ounzain S., Pedrazzini T. (2016). Super-Enhancer Lncs to Cardiovascular Development and Disease. Biochim. Et Biophys. Acta (BBA)-Mol. Cell Res..

[100] Su W., Huo Q., Wu H., Wang L., Ding X., Liang L., Zhou L., Zhao Y., Dan J., Zhang H. (2021). The Function of LncRNA-H19 in Cardiac Hypertrophy. Cell Biosci..

[101] Wolska M., Jarosz-Popek J., Junger E., Wicik Z., Porshoor T., Sharif L., Czajka P., Postula M., Mirowska-Guzel D., Czlonkowska A. (2021). Long Non-Coding RNAs as Promising Therapeutic Approach in Ischemic Stroke: A Comprehensive Review. Mol. Neurobiol..

[102] Xie L., Zhang Q., Mao J., Zhang J., Li L. (2021). The Roles of LncRNA in Myocardial Infarction: Molecular Mechanisms, Diagnosis Biomarkers, and Therapeutic Perspectives. Front. Cell Dev. Biol..

[103] Yang J., Huang X., Hu F., Fu X., Jiang Z., Chen K. (2019). LncRNA ANRIL Knockdown Relieves Myocardial Cell Apoptosis in Acute Myocardial Infarction by Regulating IL-33/ST2. Cell Cycle.

[104] Long B., Li N., Xu X.-X., Li X.-X., Xu X.-J., Guo D., Zhang D., Wu Z.-H., Zhang S.-Y. (2018). Long Noncoding RNA FTX Regulates Cardiomyocyte Apoptosis by Targeting MiR-29b-1-5p and Bcl2l2. Biochem. Biophys. Res. Commun..

[105] Cantile M., Di Bonito M., Tracey De Bellis M., Botti G. (2021). Functional Interaction among LncRNA HOTAIR and MicroRNAs in Cancer and Other Human Diseases. Cancers.

[106] Li X., Dai Y., Yan S., Shi Y., Han B., Li J., Cha L., Mu J. (2017). Down-Regulation of LncRNA KCNQ1OT1 Protects against Myocardial Ischemia/Reperfusion Injury Following Acute Myocardial Infarction. Biochem. Biophys. Res. Commun..

[107] Kumarswamy R., Bauters C., Volkmann I., Maury F., Fetisch J., Holzmann A., Lemesle G., de Groote P., Pinet F., Thum T. (2014). Circulating Long Noncoding RNA, LIPCAR, Predicts Survival in Patients With Heart Failure. Circ. Res..

[108] Chen G., Huang S., Song F., Zhou Y., He X. (2020). Lnc-Ang362 Is a pro-Fibrotic Long Non-Coding RNA Promoting Cardiac Fibrosis after Myocardial Infarction by Suppressing Smad7. Arch. Biochem. Biophys..

[109] Bu S., Singh K.K. (2021). Epigenetic Regulation of Autophagy in Cardiovascular Pathobiology. Int. J. Mol. Sci..

[110] Nukala S.B., Jousma J., Cho Y., Lee W.H., Ong S.-G. (2022). Long Non-Coding RNAs and MicroRNAs as Crucial Regulators in Cardio-Oncology. Cell Biosci..

[111] Wu H., Zhao Z.-A., Liu J., Hao K., Yu Y., Han X., Li J., Wang Y., Lei W., Dong N. (2018). Long Noncoding RNA Meg3 Regulates Cardiomyocyte Apoptosis in Myocardial Infarction. Gene Ther..

[112] Zhang J., Gao C., Meng M., Tang H. (2016). Long Noncoding RNA MHRT Protects Cardiomyocytes against H2O2-Induced Apoptosis. Biomol. Ther..

[113] Wang X., Yong C., Yu K., Yu R., Zhang R., Yu L., Li S., Cai S. (2018). Long Noncoding RNA (LncRNA) N379519 Promotes Cardiac Fibrosis in Post-Infarct Myocardium by Targeting MiR-30. Med. Sci. Monit..

[114] Magadum A., Singh N., Kurian A.A., Munir I., Mehmood T., Brown K., Sharkar M.T.K., Chepurko E., Sassi Y., Oh J.G. (2020). Pkm2 Regulates Cardiomyocyte Cell Cycle and Promotes Cardiac Regeneration. Circulation.

[115] Wang K., Liu F., Liu C.-Y., An T., Zhang J., Zhou L.-Y., Wang M., Dong Y.-H., Li N., Gao J.-N. (2016). The Long Noncoding RNA NRF Regulates Programmed Necrosis and Myocardial Injury during Ischemia and Reperfusion by Targeting MiR-873. Cell Death Differ..

[116] Micheletti R., Plaisance I., Abraham B.J., Sarre A., Ting C.-C., Alexanian M., Maric D., Maison D., Nemir M., Young R.A. (2017). The Long Noncoding RNA *Wisper* Controls Cardiac Fibrosis and Remodeling. Sci. Transl. Med..

[117] Jiao L., Li M., Shao Y., Zhang Y., Gong M., Yang X., Wang Y., Tan Z., Sun L., Xuan L. (2019). LncRNA-ZFAS1 Induces Mitochondria-Mediated Apoptosis by Causing Cytosolic Ca^2+^ Overload in Myocardial Infarction Mice Model. Cell Death Dis..

[118] Dueñas A., Expósito A., Aranega A., Franco D. (2019). The Role of Non-Coding RNA in Congenital Heart Diseases. JCDD.

[119] Lu M., Lu Q., Zhang Y., Tian G. (2011). ApoB/ApoA1 Is an Effective Predictor of Coronary Heart Disease Risk in Overweight and Obesity. J. Biomed. Res..

[120] Li X., Song F., Sun H. (2020). Long Non-coding RNA AWPPH Interacts with ROCK2 and Regulates the Proliferation and Apoptosis of Cancer Cells in Pediatric T-cell Acute Lymphoblastic Leukemia. Oncol. Lett..

[121] Li Y., Fang J., Zhou Z., Zhou Q., Sun S., Jin Z., Xi Z., Wei J. (2020). Downregulation of LncRNA BACE1-AS Improves Dopamine-Dependent Oxidative Stress in Rats with Parkinson’s Disease by Upregulating MicroRNA-34b-5p and Downregulating BACE1. Cell Cycle.

[122] Mao J., Zhou Y., Lu L., Zhang P., Ren R., Wang Y., Wang J. (2021). Identifying a Serum Exosomal-Associated LncRNA/CircRNA-MiRNA-MRNA Network in Coronary Heart Disease. Cardiol. Res. Pract..

[123] Hennessy E.J., van Solingen C., Scacalossi K.R., Ouimet M., Afonso M.S., Prins J., Koelwyn G.J., Sharma M., Ramkhelawon B., Carpenter S. (2019). The Long Noncoding RNA CHROME Regulates Cholesterol Homeostasis in Primates. Nat. Metab..

[124] Guo F., Sha Y., Hu B., Li G. (2021). Correlation of Long Non-Coding RNA LncRNA-FA2H-2 With Inflammatory Markers in the Peripheral Blood of Patients With Coronary Heart Disease. Front. Cardiovasc. Med..

[125] Toni L., Hailu F., Sucharov C.C. (2020). Dysregulated Micro-RNAs and Long Noncoding RNAs in Cardiac Development and Pediatric Heart Failure. Am. J. Physiol.-Heart Circ. Physiol..

[126] Huang Y., Wang L., Mao Y., Nan G. (2019). Long Noncoding RNA-H19 Contributes to Atherosclerosis and Induces Ischemic Stroke via the Upregulation of Acid Phosphatase 5. Front. Neurol..

[127] Sun Y., Huang S., Wan C., Ruan Q., Xie X., Wei D., Li G., Lin S., Li H., Wu S. (2021). Knockdown of LncRNA ENST00000609755.1 Confers Protection Against Early OxLDL-Induced Coronary Heart Disease. Front. Cardiovasc. Med..

[128] Wang F., Cai X., Jiao P., Liu Y., Yuan B., Zhang P., Liu H., Ma L. (2020). Relationship between Long Non-Coding RNA and Prognosis of Patients with Coronary Heart Disease after Percutaneous Coronary Intervention: A Protocol for Systematic Review and Meta-Analysis. Medicine.

[129] Wu G., Cai J., Han Y., Chen J., Huang Z.-P., Chen C., Cai Y., Huang H., Yang Y., Liu Y. (2014). LincRNA-P21 Regulates Neointima Formation, Vascular Smooth Muscle Cell Proliferation, Apoptosis, and Atherosclerosis by Enhancing P53 Activity. Circulation.

[130] Wang Q.-C., Wang Z.-Y., Xu Q., Chen X.-L., Shi R.-Z. (2021). LncRNA Expression Profiles and Associated CeRNA Network Analyses in Epicardial Adipose Tissue of Patients with Coronary Artery Disease. Sci. Rep..

[131] Cao C., Zhen W., Yu H., Zhang L., Liu Y. (2021). LncRNA MALAT1/MiR-143 Axis Is a Potential Biomarker for in-Stent Restenosis and Is Involved in the Multiplication of Vascular Smooth Muscle Cells. Open Life Sci..

[132] Saygili H., Bozgeyik I., Yumrutas O., Akturk E., Bagis H. (2021). Differential Expression of Long Noncoding RNAs in Patients with Coronary Artery Disease. Mol. Syndr..

[133] Hu Y.-W., Guo F.-X., Xu Y.-J., Li P., Lu Z.-F., McVey D.G., Zheng L., Wang Q., Ye J.H., Kang C.-M. (2019). Long Noncoding RNA NEXN-AS1 Mitigates Atherosclerosis by Regulating the Actin-Binding Protein NEXN. J. Clin. Investig..

[134] Liao J., Wang J., Liu Y., Li J., Duan L. (2019). Transcriptome Sequencing of LncRNA, MiRNA, MRNA and Interaction Network Constructing in Coronary Heart Disease. BMC Med. Genom..

[135] Jin L., Lin X., Yang L., Fan X., Wang W., Li S., Li J., Liu X., Bao M., Cui X. (2018). AK098656, a Novel Vascular Smooth Muscle Cell–Dominant Long Noncoding RNA, Promotes Hypertension. Hypertension.

[136] Gholami L., Ghafouri-Fard S., Mirzajani S., Arsang-Jang S., Taheri M., Dehbani Z., Dehghani S., Houshmand B., Amid R., Sayad A. (2020). The LncRNA ANRIL Is Down-Regulated in Peripheral Blood of Patients with Periodontitis. Non-Coding RNA Res..

[137] Luo Y., Guo J., Xu P., Gui R. (2020). Long Non-Coding RNA GAS5 Maintains Insulin Secretion by Regulating Multiple MiRNAs in INS-1 832/13 Cells. Front. Mol. Biosci..

[138] Das S., Zhang E., Senapati P., Amaram V., Reddy M.A., Stapleton K., Leung A., Lanting L., Wang M., Chen Z. (2018). A Novel Angiotensin II–Induced Long Noncoding RNA *Giver* Regulates Oxidative Stress, Inflammation, and Proliferation in Vascular Smooth Muscle Cells. Circ. Res..

[139] Yu B., Wang S. (2018). Angio-LncRs: LncRNAs That Regulate Angiogenesis and Vascular Disease. Theranostics.

[140] Jusic A., Devaux Y. (2019). On behalf of the EU-CardioRNA COST Action (CA17129) Noncoding RNAs in Hypertension. Hypertension.

[141] Han Y., Ali M.K., Dua K., Spiekerkoetter E., Mao Y. (2021). Role of Long Non-Coding RNAs in Pulmonary Arterial Hypertension. Cells.

[142] El Azzouzi H., Doevendans P.A., Sluijter J.P.G. (2016). Long Non-Coding RNAs in Heart Failure: An Obvious Lnc. Ann. Transl. Med..

[143] Greco S., Zaccagnini G., Fuschi P., Voellenkle C., Carrara M., Sadeghi I., Bearzi C., Maimone B., Castelvecchio S., Stellos K. (2017). Increased BACE1-AS Long Noncoding RNA and β-Amyloid Levels in Heart Failure. Cardiovasc. Res..

[144] Ottaviani L., Martins P.A.D.C. (2017). Non-coding RNAs in cardiac hypertrophy. J. Physiol..

[145] Gomes C.P.d.C., Schroen B., Kuster G.M., Robinson E.L., Ford K., Squire I.B., Heymans S., Martelli F., Emanueli C., Devaux Y. (2020). Regulatory RNAs in Heart Failure. Circulation.

[146] Fan J., Li H., Xie R., Zhang X., Nie X., Shi X., Zhan J., Yin Z., Zhao Y., Dai B. (2021). LncRNA ZNF593-AS Alleviates Contractile Dysfunction in Dilated Cardiomyopathy. Circ. Res..

[147] Wang S., Lv T., Chen Q., Yang Y., Xu L., Zhang X., Wang E., Hu X., Liu Y. (2022). Transcriptome Sequencing and LncRNA-MiRNA-MRNA Network Construction in Cardiac Fibrosis and Heart Failure. Bioengineered.

[148] Greco S., Zaccagnini G., Perfetti A., Fuschi P., Valaperta R., Voellenkle C., Castelvecchio S., Gaetano C., Finato N., Beltrami A.P. (2016). Long Noncoding RNA Dysregulation in Ischemic Heart Failure. J. Transl. Med..

[149] Santer L., López B., Ravassa S., Baer C., Riedel I., Chatterjee S., Moreno M.U., González A., Querejeta R., Pinet F. (2019). Circulating Long Noncoding RNA LIPCAR Predicts Heart Failure Outcomes in Patients Without Chronic Kidney Disease. Hypertension.

[150] Sato M., Kadomatsu T., Miyata K., Warren J.S., Tian Z., Zhu S., Horiguchi H., Makaju A., Bakhtina A., Morinaga J. (2021). The LncRNA Caren Antagonizes Heart Failure by Inactivating DNA Damage Response and Activating Mitochondrial Biogenesis. Nat. Commun..

[151] Pinheiro A., Naya F.J. (2021). The Key Lnc (RNA)s in Cardiac and Skeletal Muscle Development, Regeneration, and Disease. J. Cardiovasc. Dev. Dis..

[152] Han P., Chang C.-P. (2015). Long Non-Coding RNA and Chromatin Remodeling. RNA Biol..

[153] Yang L., Deng J., Ma W., Qiao A., Xu S., Yu Y., Boriboun C., Kang X., Han D., Ernst P. (2021). Ablation of LncRNA *Miat* Attenuates Pathological Hypertrophy and Heart Failure. Theranostics.

[154] Zheng Y., Zhang Y., Zhang X., Dang Y., Cheng Y., Hua W., Teng M., Wang S., Lu X. (2021). Novel LncRNA-MiRNA-MRNA Competing Endogenous RNA Triple Networks Associated Programmed Cell Death in Heart Failure. Front. Cardiovasc. Med..

[155] Garcia-Padilla C., Lozano-Velasco E., Garcia-Lopez V., Aranega A., Franco D., Garcia-Martinez V., Lopez-Sanchez C. (2022). Comparative Analysis of Non-Coding RNA Transcriptomics in Heart Failure. Biomedicines.

[156] Ou Y., Liao C., Li H., Yu G. (2020). LncRNA SOX2OT/Smad3 Feedback Loop Promotes Myocardial Fibrosis in Heart Failure. IUBMB Life.

[157] Di Salvo T.G., Guo Y., Su Y.R., Clark T., Brittain E., Absi T., Maltais S., Hemnes A. (2015). Right Ventricular Long Noncoding RNA Expression in Human Heart Failure. Pulm. Circ..

[158] Jaminon A., Reesink K., Kroon A., Schurgers L. (2019). The Role of Vascular Smooth Muscle Cells in Arterial Remodeling: Focus on Calcification-Related Processes. Int. J. Mol. Sci..

[159] Wu G., Jose P.A., Zeng C. (2018). Noncoding RNAs in the Regulatory Network of Hypertension. Hypertension.

[160] Zhou H., Wang B., Yang Y., Jia Q., Zhang A., Qi Z., Zhang J. (2019). Long Noncoding RNAs in Pathological Cardiac Remodeling: A Review of the Update Literature. BioMed Res. Int..

[161] Lu B.-H., Liu H.-B., Guo S.-X., Zhang J., Li D.-X., Chen Z.-G., Lin F., Zhao G.-A. (2022). Long Non-Coding RNAs: Modulators of Phenotypic Transformation in Vascular Smooth Muscle Cells. Front. Cardiovasc. Med..

[162] Libby P., Buring J.E., Badimon L., Hansson G.K., Deanfield J., Bittencourt M.S., Tokgözoğlu L., Lewis E.F. (2019). Atherosclerosis. Nat. Rev. Dis. Prim..

[163] Archer K., Broskova Z., Bayoumi A.S., Teoh J.-p., Davila A., Tang Y., Su H., Kim I.-m. (2015). Long Non-Coding RNAs as Master Regulators in Cardiovascular Diseases. Int. J. Mol. Sci..

[164] Wysoczynski M., Kim J., Moore J.B., Uchida S. (2020). Macrophage Long Non-Coding RNAs in Pathogenesis of Cardiovascular Disease. Non-Coding RNA.

[165] Vausort M., Wagner D.R., Devaux Y. (2014). Long Noncoding RNAs in Patients With Acute Myocardial Infarction. Circ. Res..

[166] Hermans-Beijnsberger S., van Bilsen M., Schroen B. (2018). Long Non-Coding RNAs in the Failing Heart and Vasculature. Non-Coding RNA Res..

[167] Beijnsberger S. (2019). Beijnsberger Emerging Roles of Small and Long Non-Coding RNAs in Cardiac Disease.

[168] Fan X., Weng X., Zhao Y., Chen W., Gan T., Xu D. (2017). Circular RNAs in Cardiovascular Disease: An Overview. BioMed Res. Int..

[169] Tang Y., Bao J., Hu J., Liu L., Xu D. (2021). Circular RNA in Cardiovascular Disease: Expression, Mechanisms and Clinical Prospects. J. Cell. Mol. Med..

[170] Holdt L.M., Kohlmaier A., Teupser D. (2018). Molecular Functions and Specific Roles of CircRNAs in the Cardiovascular System. Non-Coding RNA Res..

[171] Holdt L.M., Stahringer A., Sass K., Pichler G., Kulak N.A., Wilfert W., Kohlmaier A., Herbst A., Northoff B.H., Nicolaou A. (2016). Circular Non-Coding RNA ANRIL Modulates Ribosomal RNA Maturation and Atherosclerosis in Humans. Nat. Commun..

[172] Sygitowicz G., Sitkiewicz D. (2022). Involvement of CircRNAs in the Development of Heart Failure. Int. J. Mol. Sci..

[173] Sun C., Ni M., Song B., Cao L. (2020). Circulating Circular RNAs: Novel Biomarkers for Heart Failure. Front. Pharmacol..

[174] Prestes P.R., Maier M.C., Woods B.A., Charchar F.J. (2020). A Guide to the Short, Long and Circular RNAs in Hypertension and Cardiovascular Disease. Int. J. Mol. Sci..

[175] Wu S., Chen L., Zhou X. (2022). Circular RNAs in the Regulation of Cardiac Hypertrophy. Mol. Ther.-Nucleic Acids.

[176] Lim T.B., Aliwarga E., Luu T.D.A., Li Y.P., Ng S.L., Annadoray L., Sian S., Ackers-Johnson M.A., Foo R.S.-Y. (2019). Targeting the Highly Abundant Circular RNA CircSlc8a1 in Cardiomyocytes Attenuates Pressure Overload Induced Hypertrophy. Cardiovasc. Res..

[177] Li H., Xu J.-D., Fang X.-H., Zhu J.-N., Yang J., Pan R., Yuan S.-J., Zeng N., Yang Z.-Z., Yang H. (2020). Circular RNA CircRNA_000203 Aggravates Cardiac Hypertrophy via Suppressing MiR-26b-5p and MiR-140-3p Binding to Gata4. Cardiovasc. Res..

[178] Pan J., Xu Z., Guo G., Xu C., Song Z., Li K., Zhong K., Wang D. (2021). Circ_nuclear Factor I X (CircNfix) Attenuates Pressure Overload-Induced Cardiac Hypertrophy via Regulating MiR-145-5p/ATF3 Axis. Bioengineered.

[179] Li J., Han Y., Wang S., Wu X., Cao J., Sun T. (2023). Circular RNAs: Biogenesis, Biological Functions, and Roles in Myocardial Infarction. Int. J. Mol. Sci..

[180] Cai L., Qi B., Wu X., Peng S., Zhou G., Wei Y., Xu J., Chen S., Liu S. (2019). Circular RNA Ttc3 Regulates Cardiac Function after Myocardial Infarction by Sponging MiR-15b. J. Mol. Cell. Cardiol..

[181] Wang Y., Liu B. (2020). Circular RNA in Diseased Heart. Cells.

[182] Chen Y., Zhou J., Wei Z., Cheng Y., Tian G., Quan Y., Kong Q., Wu W., Liu X. (2022). Identification of Circular RNAs in Cardiac Hypertrophy and Cardiac Fibrosis. Front. Pharmacol..

[183] Jiang L., Wang X., Zhan X., Kang S., Liu H., Luo Y., Lin L. (2020). Advance in Circular RNA Modulation Effects of Heart Failure. Gene.

[184] Zhang L., Wang Y., Yu F., Li X., Gao H., Li P. (2021). CircHIPK3 Plays Vital Roles in Cardiovascular Disease. Front. Cardiovasc. Med..

[185] Tang L., Li P., Jang M., Zhu W. (2021). Circular RNAs and Cardiovascular Regeneration. Front. Cardiovasc. Med..

[186] Wen Z.-J., Xin H., Wang Y.-C., Liu H.-W., Gao Y.-Y., Zhang Y.-F. (2021). Emerging Roles of CircRNAs in the Pathological Process of Myocardial Infarction. Mol. Ther.-Nucleic Acids.

[187] Shi P., Ji H., Zhang H., Yang J., Guo R., Wang J. (2020). CircANRIL Reduces Vascular Endothelial Injury, Oxidative Stress and Inflammation in Rats with Coronary Atherosclerosis. Exp. Ther. Med..

[188] Gao X., Tian X., Huang Y., Fang R., Wang G., Li D., Zhang J., Li T., Yuan R. (2022). Role of Circular RNA in Myocardial Ischemia and Ageing-Related Diseases. Cytokine Growth Factor Rev..

[189] Du W.W., Yang W., Chen Y., Wu Z.-K., Foster F.S., Yang Z., Li X., Yang B.B. (2016). Foxo3 Circular RNA Promotes Cardiac Senescence by Modulating Multiple Factors Associated with Stress and Senescence Responses. Eur. Heart J..

[190] Yang T., Long T., Du T., Chen Y., Dong Y., Huang Z.-P. (2021). Circle the Cardiac Remodeling With CircRNAs. Front. Cardiovasc. Med..

[191] Wu W., Zhou M., Liu D., Min X., Shao T., Xu Z., Jing X., Cai M., Xu S., Liang X. (2021). CircGNAQ, a Circular RNA Enriched in Vascular Endothelium, Inhibits Endothelial Cell Senescence and Atherosclerosis Progression. Mol. Ther.-Nucleic Acids.

[192] Garikipati V.N.S., Verma S.K., Cheng Z., Liang D., Truongcao M.M., Cimini M., Yue Y., Huang G., Wang C., Benedict C. (2019). Circular RNA CircFndc3b Modulates Cardiac Repair after Myocardial Infarction via FUS/VEGF-A Axis. Nat. Commun..

[193] Min X., Liu D., Xiong X. (2021). Circular RNAs as Competing Endogenous RNAs in Cardiovascular and Cerebrovascular Diseases: Molecular Mechanisms and Clinical Implications. Front. Cardiovasc. Med..

[194] Pan L., Lian W., Zhang X., Han S., Cao C., Li X., Li M. (2018). Human Circular RNA-0054633 Regulates High Glucose-induced Vascular Endothelial Cell Dysfunction through the MicroRNA-218/Roundabout 1 and MicroRNA-218/Heme Oxygenase-1 Axes. Int. J. Mol. Med..

[195] Chen J., Cui L., Yuan J., Zhang Y., Sang H. (2017). Circular RNA WDR77 Target FGF-2 to Regulate Vascular Smooth Muscle Cells Proliferation and Migration by Sponging MiR-124. Biochem. Biophys. Res. Commun..

[196] Sun J., Zhang Z., Yang S. (2019). Circ_RUSC2 Upregulates the Expression of MiR-661 Target Gene *SYK* and Regulates the Function of Vascular Smooth Muscle Cells. Biochem. Cell Biol..

[197] Turer A.T., Hill J.A. (2010). Pathogenesis of Myocardial Ischemia-Reperfusion Injury and Rationale for Therapy. Am. J. Cardiol..

[198] Zhao Q., Li W., Pan W., Wang Z. (2021). CircRNA 010567 Plays a Significant Role in Myocardial Infarction via the Regulation of the MiRNA-141/DAPK1 Axis. J. Thorac. Dis..

[199] Zhang S., Wang W., Wu X., Zhou X. (2020). Regulatory Roles of Circular RNAs in Coronary Artery Disease. Mol. Ther.-Nucleic Acids.

[200] Morbach C., Wagner M., Güntner S., Malsch C., Oezkur M., Wood D., Kotseva K., Leyh R., Ertl G., Karmann W. (2017). Heart Failure in Patients with Coronary Heart Disease: Prevalence, Characteristics and Guideline Implementation—Results from the German EuroAspire IV Cohort. BMC Cardiovasc. Disord..

[201] Si X., Zheng H., Wei G., Li M., Li W., Wang H., Guo H., Sun J., Li C., Zhong S. (2020). CircRNA Hipk3 Induces Cardiac Regeneration after Myocardial Infarction in Mice by Binding to Notch1 and MiR-133a. Mol. Ther.-Nucleic Acids.

[202] Schultheiss H.-P., Fairweather D., Caforio A.L.P., Escher F., Hershberger R.E., Lipshultz S.E., Liu P.P., Matsumori A., Mazzanti A., McMurray J. (2019). Dilated Cardiomyopathy. Nat. Rev. Dis. Prim..

[203] Long Q., Lv B., Jiang S., Lin J. (2023). The Landscape of Circular RNAs in Cardiovascular Diseases. Int. J. Mol. Sci..

[204] Mester-Tonczar J., Hašimbegović E., Spannbauer A., Traxler D., Kastner N., Zlabinger K., Einzinger P., Pavo N., Goliasch G., Gyöngyösi M. (2020). Circular RNAs in Cardiac Regeneration: Cardiac Cell Proliferation, Differentiation, Survival, and Reprogramming. Front. Physiol..

[205] Jiapaer Z., Li C., Yang X., Sun L., Chatterjee E., Zhang L., Lei J., Li G. (2023). Extracellular Non-Coding RNAs in Cardiovascular Diseases. Pharmaceutics.

[206] Shah A.M., Giacca M. (2022). Small non-coding RNA therapeutics for cardiovascular disease. Eur. Heart J..

[207] Braga L., Ali H., Secco I., Giacca M. (2021). Non-coding RNA therapeutics for cardiac regeneration. Non-coding RNA therapeutics for cardiac regeneration. Cardiovasc. Res..

[208] Boon R.A., Iekushi K., Lechner S., Seeger T., Fischer A., Heydt S., Kaluza D., Tréguer K., Carmona G., Bonauer A. (2013). MicroRNA-34a regulates cardiac ageing and function. Nature.

[209] Gandhi S., Ruehle F., Stoll M. (2019). Evolutionary Patterns of Non-Coding RNA in Cardiovascular Biology. Non-Coding RNA.

